# Effects of Grape Seed Extract and Proanthocyanidin B2 on In Vitro Proliferation, Viability, Steroidogenesis, Oxidative Stress, and Cell Signaling in Human Granulosa Cells

**DOI:** 10.3390/ijms20174215

**Published:** 2019-08-28

**Authors:** Alix Barbe, Christelle Ramé, Namya Mellouk, Anthony Estienne, Alice Bongrani, Adeline Brossaud, Antonella Riva, Fabrice Guérif, Pascal Froment, Joëlle Dupont

**Affiliations:** 1 INRA, UMR 85 Physiologie de la Reproduction et des Comportements, F-37380 Nouzilly, France; 2 CNRS, UMR 7247 Physiologie de la Reproduction et des Comportements, F-37380 Nouzilly, France; 3 Department of Animal Physiology, University of François Rabelais, F-37041 Tours, France; 4 Institut Français du Cheval et de l'Equitation, F-37380 Nouzilly, France; 5 INDENA S.p.A, 12-20139 Milan, Italy; 6 Service de Médecine et Biologie de la Reproduction, Hospital of Tours, F-37044 Tours, France

**Keywords:** human granulosa cells, grape seed extract, oxidative stress, steroidogenesis, cell signaling, apoptosis

## Abstract

Reactive oxygen species (ROS) which lead to oxidative stress affect ovarian function. Grape seed extract (GSE) could be proposed as an effective antioxidant, particularly due to its proanthocyanidin content. In this study, we investigated a dose effect (0, 0.01, 0.1, 1, 10, 50, and 100 μg/mL) of GSE and proanthocyanidin B2 (GSPB2) on the ROS content, cell proliferation, cell viability, and steroidogenesis in both primary luteinized granulosa cells (hGC) and the tumor granulosa cell line (KGN). The levels of ROS were measured using ROS-Glo assay. Cell proliferation and viability were evaluated by [3H]-thymidine incorporation and Cell Counting Kit-8 (CCK8) assay, respectively. Steroid secretion was evaluated by radioimmunoassay. We also analyzed the cell cycle component protein level and signaling pathways by immunoblot and the NOX4 mRNA expression by RTqPCR. From 0.1 to 1 μg/mL, GSE and GSBP2 reduced the ROS cell content and the NOX4 mRNA levels, whereas, GSE and GSBP2 increased the ROS cell content from 50 to 100 μM in both hGC and KGN. GSE and GSPB2 treatments at 50 and 100 μg/mL induced a delay in G_1_ to S phase cell cycle progression as determined by fluorescence-activated cell sorting. Consequently, they reduced cell growth, cyclin D2 amount, and Akt phosphorylation, and they increased protein levels of p21 and p27 cyclin-dependent kinase inhibitors. These data were also associated with an increase in cell death that could be due to a reduction in Bcl-2-associated death promoter (BAD) phosphorylation and an increase in the cleaved-caspase-3 level. All these negative effects were not observed at lower concentrations of GSE and GSPB2 (0.01 to 10 μg/mL). Interestingly, we found that GSE and GSPB2 treatments (0.1 to 100 μg/mL) improved progesterone and estradiol secretion and this was associated with a higher level of the cholesterol carriers, StAR (steroidogenic acute regulatory protein), CREB (Cyclic adenosine monophosphate Response Element-binding protein), and MAPK ERK1/2 (Mitogen-Activated Protein Kinases Extracellular signal-Regulated Kinases 1/2) phosphorylation in both hGC and KGN cells. Taken together, GSE and GSPB2 (0.1–10 μg/mL) in vitro treatments decrease oxidative stress and increase steroidogenesis without affecting cell proliferation and viability in human granulosa cells.

## 1. Introduction

The ovarian processes are regulated by various hormonal signaling molecules that are themselves controlled by several physiological regulators including reactive oxygen species (ROS) [1,2]. ROS are produced in the ovarian follicle and corpus luteum, and they play crucial roles in follicular development and survival [3]. The effects of oxidative stress on female reproduction have been widely described [4]. It is also well known that ROS are important molecules involved in the regulation of cell signaling when maintained at physiological cellular concentrations. For example, superoxide which is generated by NADPH oxidases (NOX) comes from cell metabolism [5]. However, an excess of ROS production can induce oxidative damage [6,7]. All non-pathological cells are equipped with a complex antioxidant defense system to counterbalance the effect of oxidants [8].

In rat ovary, the beneficial roles of the ROS level have been demonstrated, including the meiotic resumption and maturation of oocytes [9,10,11]. However, it has also been reported that an accumulation of ROS leads to oxidative stress, deteriorates oocyte quality, and induces granulosa cell apoptosis [12,13,14,15]. Furthermore, it reduces granulosa-oocyte communication affecting the quality of preovulatory oocytes. The role of ROS in gynecological diseases and assisted reproduction has been widely studied in recent years [16]. The role of ROS concentrations in the follicular fluid for successful in vitro fertilization (IVF) outcome is contradictory [17]. In humans, an increased percentage of ROS producing granulosa cells results in fewer oocytes retrieved and diminishes the implantation rate [18]. However, environmental and lifestyle changes (psychological stress, cigarette smoking, alcohol consumption, and environmental exposures) and pathological conditions can induce accumulation of ROS leading to oxidative stress that may affect female reproductive health. Indeed, reports suggest that chronic psychological stress results in a poor IVF outcome, possibly due to its negative impact on the level of ROS in the ovary and oocytes [19,20]. Furthermore, endocrine disruptors may cause ROS accumulation, DNA damages, and an increase in oxidative stress in mouse oocyte [21], as well as the repeated ovarian stimulation by exogenous gonadotropin induces oxidative stress in the ovary and poor quality ovulation in mice [22]. 

Several attempts have been carried out to use antioxidant supplementation as a means of counteracting the oxidative stress conditions by restoring the cellular antioxidant defense mechanism. Grape seed extract (GSE) from seeds of Vitis vinifera contains proanthocyanidin that possesses a wide range of biological activities such as antioxidant, anticarcinogenic, and anti-inflammatory effects [23,24,25,26,27]. The grape seed proanthocyanidin extracts (GSPE) are mostly dimers, trimers, and highly polymerized oligomers of monomeric catechins [26,28]. Dimeric proanthocyanidin B2 (GSPB2) is one of the most important components of GSPE that exhibits protective effects against stress, inflammation, and cardiovascular diseases [29]. GSPE are also potent antioxidants and free radical scavengers, being more effective than either ascorbic acid or vitamin E [30]. In in vivo experiments, the GSPE treatment can prevent the ovarian aging process in hens by reducing oxidative stress [31]. In mice, GSPB2 exerts a potent and beneficial role in reducing granulosa cell apoptosis and inducing the autophagy process [32]. However, no studies have investigated the effects of GSE and GSPB2 on human ovarian cells and, more precisely, on human granulosa cells. Therefore, the objective of our study was to determine the effect of various concentrations of GSE and GSPB2 on cell proliferation, viability, steroidogenesis, and oxidative stress in both the tumor cell line of human granulosa cells (KGN) and primary luteinized human granulosa cells (hGC).

## 2. Results

### 2.1. Effect of GSE and GSPB2 Treatments on Cell Proliferation in Human Granulosa Cells

**Figure 1:**
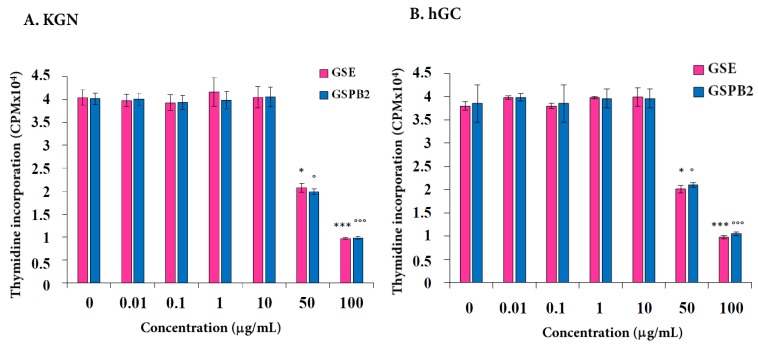
Effect of grape seed extract (GSE) and dimeric proanthocyanidin B2 (GSPB2) treatments on cell proliferation in human granulosa cells. Thymidine incorporation was determined in human granulosa cells (KGN) (**A**) and primary luteinized human granulosa cells (hGC) (**B**) cultured for 24 hours in appropriate medium with 10% FBS supplemented with or without different concentrations of GSE and GSPB2 (0, 0.01, 0.1, 1, 10, 50, and 100 μg/mL). CPM: counts per minute. Results are represented as mean ± SEM. The results are representative of four independent cultures with each condition in quadruplet. For the hGC cells, an independent culture was from a pool of cells collected from three patients. * and *** represent a significant effect of GSE as compared to the control at *p* ≤ 0.05 and *p* ≤ 0.001, respectively. ° and °°° represent a significant effect of GSPB2 as compared to the control at *p* ≤ 0.05 and *p* ≤ 0.001, respectively.

We determined the effect of different concentrations of GSE and GSPB2 (0.01 to 100 μg/mL) on the [3H]-thymidine incorporation level in KGN and hGC after 24 hours of treatment. As shown in Figure 1A (KGN) and Figure 1B (hGC), the [3H]-thymidine incorporation level was not affected by the GSE and GSPB2 treatments until a concentration of 10 μg/mL, whereas, it was significantly reduced by about two- and three-fold in response to 50 μg/mL (Figure 1A (KGN), GSE *p* = 0.02 and GSPB2 *p* = 0.01; Figure 1B (hGC), GSE *p* = 0.03 and GSPB2 *p* = 0.01) and 100 μg/mL (Figure 1A (KGN), GSE *p* = 0.001 and GSPB2 *p* = 0.001; Figure 1B (hGC), GSE *p* = 0.001 and GSPB2 *p* = 0.001), respectively, in both cell types as compared to the control (no GSE or GSPB2 treatment). 

### 2.2. G1 Growth Arrest in Response to GSE and GSPB2 Treatments in Human Granulosa Cells

FACS scan analysis was used to determine the effects of GSE and GSPB2 treatments on the distribution of KGN cells through the phases of the cell cycle in response to various concentrations of GSE and GSPB2. As shown in Table 1, 31.6%, 29.7%, 29.7%, 19.4%, and 11.9% of KGN cells incubated for 24 h with GSE at 0, 1, 10, 50, and 100 μg/mL, respectively, progressed through S phase, whereas, 55%, 56%, 76%, and 84% of cells remained in the G0–G1 stages. Similar results were obtained with the GSPB2 treatment (Table 1). Thus, the decrease in proliferation rate in response to GSE and GSPB2 at 50 and 100 μg/mL in KGN cells is associated with an increase in the proportion of cells in G1 suggesting a delay in G1 to S progression.

### 2.3. Expression of Cyclin D2 and Cyclin-Dependent Kinase Inhibitors p21 and p27 in Response to GSE and GSPB2 Treatments in Human Granulosa Cells

**Figure 2:**
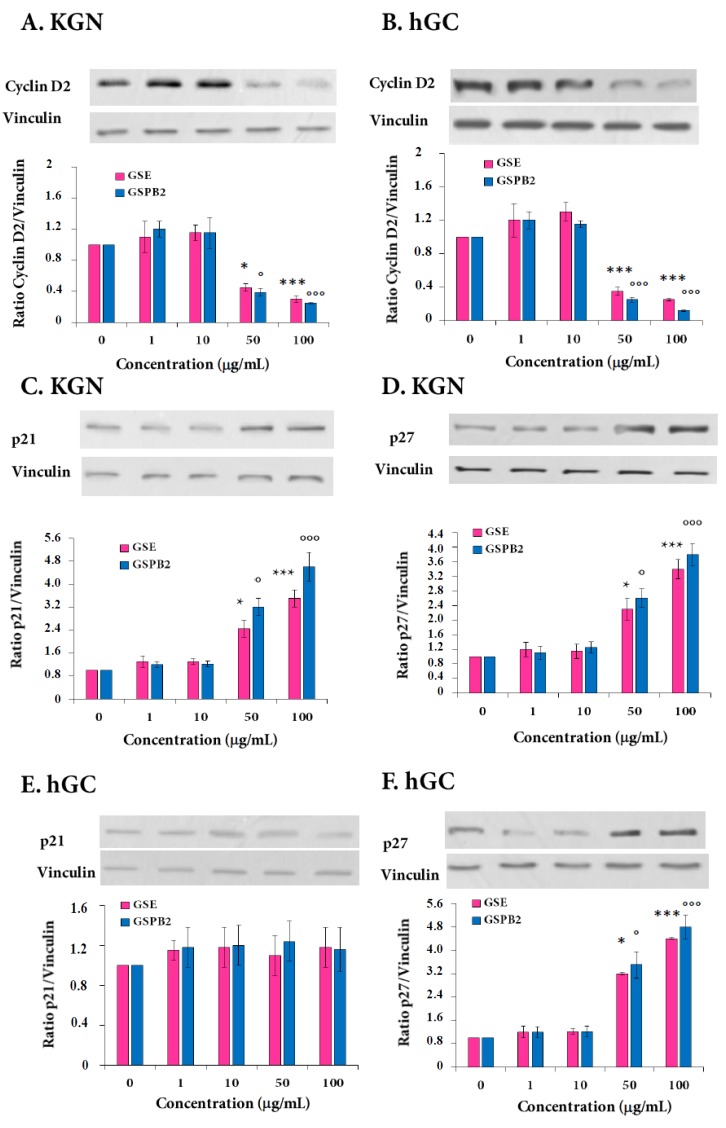
Effect of the GSE and GSPB2 treatments on the cyclin D2 (**A**,**B**), cyclin-dependent kinase inhibitors p21 (**C**,**D**) and p27 (**E**,**F**) protein levels in human granulosa cells. Human granulosa cells (KGN) (**A**,**C**,**D**) and hGC (**B**,**E**,**F**) were cultured in appropriate medium with 10% FBS and then supplemented for 24 h (cyclin D2, **A**,**B**) and (CDKIs p21 and p27, **C**–**F**) with different concentrations of GSE and GSPB2 (0, 1, 10, 50, and 100 μg/mL). Cells were then harvested and cyclin D2, p21, and p27 protein levels were determined by immunoblot. Samples contained an equal amount of protein, as confirmed by reprobing each membrane with an anti-vinculin antibody. In each panel, immunoreactivity was quantified as described in Materials and Methods, and the ratio cyclin D2, CDKI p21 and p27 /vinculin were represented. Representative blots in response to different concentrations of GSE are shown. The results are means ± SEM of four independent cultures. For hGC cells, an independent culture was from a pool of cells collected from three patients. * and *** represent a significant effect of GSE as compared to the control at *p* ≤ 0.05 and *p* ≤ 0.001, respectively. ° and °°° represent a significant effect of GSPB2 as compared to the control at *p* ≤ 0.05 and *p* ≤ 0.001, respectively.

In order to explain further the G1 arrest in human granulosa cells in response to the GSE and GSPB2 treatments, we studied some cell cycle components by Western blot. As shown in Figure 2A,B and Supplemental Figures S1 and S2, the GSE and GSPB2 treatments (50 and 100 μg/mL) reduced significantly cyclin D2 protein levels (Figure 2A, GSE and GSPB2 (50 μg/mL) as compared to control *p* = 0.04 and *p* = 0.02, respectively and both GSE and GSPB2 (100 μg/ml) as compared to control *p* < 0.001; Figure 2B, both GSE and GSPB2 (50 and 100 μg/ml) *p* < 0.001). We have also shown that the GSE treatment (50 and 100 μg/mL) increased significantly the protein levels of the cyclin-dependent kinase inhibitors (CDKIs) p21 and p27 (Figure 2C and Supplemental Figures S3–S6, GSE and GSPB2 (50 μg/mL) as compared to control *p* = 0.03 and *p* = 0.01, respectively, and both GSE and GSPB2 (100 μg/mL) as compared to control *p* < 0.001; and Figure 2D and Supplemental Figure S5, GSE and GSPB2 (50 μg/mL) as compared to control *p* = 0.04 and *p* = 0.02, respectively, and both GSE and GSPB2 (100 μg/mL) as compared to control *p* < 0.001) in KGN cells, whereas, they induced only an increase in p27 protein levels in hGC cells (Figure 2E,F and Supplemental Figures S4 and S6, GSE and GSPB2 (50 μg/mL) as compared to control *p* = 0.01 and *p* = 0.03, respectively, and both GSE and GSPB2 (100 μg/mL) as compared to control *p* < 0.001). Thus, the G1 arrest in response to GSE and GSPB2 is due at least to some extent to the reduction of cyclin D2 expression and to the increase of levels of CDKIs p21 and p27.

### 2.4. Effect of GSE and GSPB2 Treatments on Cell Viability and Apoptosis in Human Granulosa Cells

**Figure 3:**
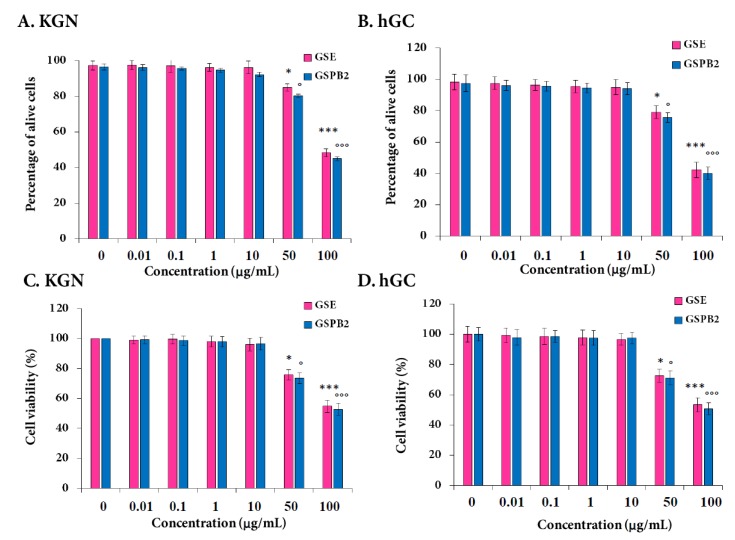
Effect of the GSE and GSPB2 treatments on the number of alive cells (**A**,**B**) and cell viability (**C**,**D**) in human granulosa cells. KGN (**A**,**C**) and hGC (**B**,**D**) cells were plated and incubated in various concentrations of GSE and GSPB2 (0, 0.01, 0.1, 1, 10, 50, and 100 μg/mL for 24 h in appropriate medium with serum. Then, cell viability was measured by trypan blue staining (**A**,**B**) and Cell Counting Kit-8 (CCK8 reagent, **C**,**D**) as described in Materials and Methods. The results are means ± SEM of four independent cultures with each condition in quadruplet. For the hGC cells, an independent culture was from a pool of cells collected from three patients. * and *** represent a significant effect of GSE as compared to the control at *p* ≤ 0.05 and *p* ≤ 0.001, respectively. ° and °°° represent a significant effect of GSPB2 as compared to the control at *p* ≤ 0.05 and *p* ≤ 0.001, respectively.

Trypan blue staining demonstrated that both GSE and GSPB2 (50 and 100 μg/ml, 24 h) treatments reduced by about 20% and 50% the number of alive KGN and hGC cells (Figure 3A, GSE and GSPB2 (50 μg/mL) as compared to control *p* = 0.05 and *p* = 0.03, respectively, and both GSE and GSPB2 (100 μg/mL) as compared to control *p* < 0.001; and Figure 3B, GSE and GSPB2 (50 μg/mL) as compared to control *p* = 0.05 and *p* = 0.03, respectively, and both GSE and GSPB2 (100 μg/mL) as compared to control *p* < 0.001). Similar results were obtained on the cell viability by using the CCK-8 assay (Figure 3C, GSE and GSPB2 (50 μg/mL) as compared to control *p* = 0.05 and *p* = 0.03, respectively, and both GSE and GSPB2 (100 μg/mL) as compared to control *p* < 0.001; and Figure 3D, both GSE and GSPB2 (50 μg/mL) as compared to control *p* = 0.01, and both GSE and GSPB2 (100 μg/mL) as compared to control *p* < 0.001).

**Figure 4:**
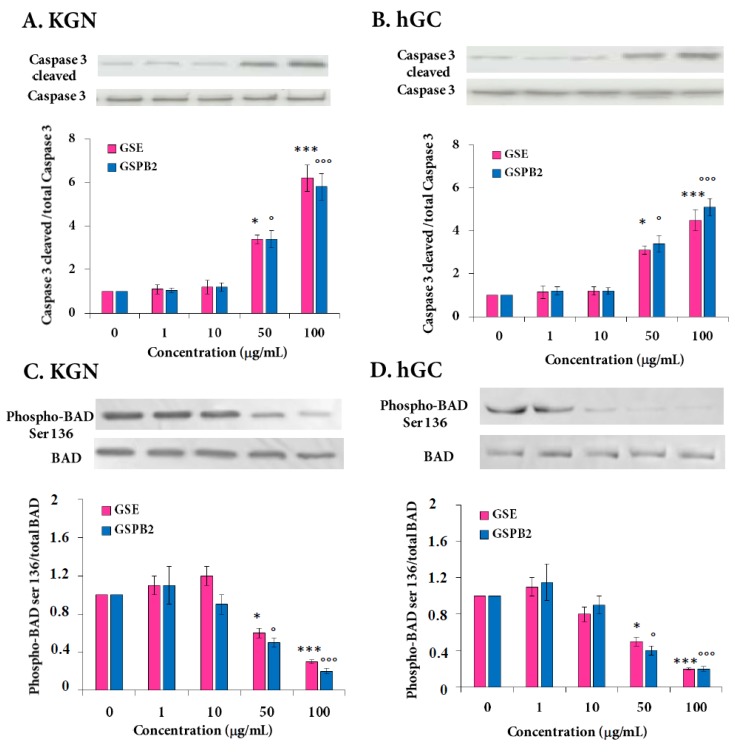
Effect of the GSE and GSPB2 treatments on the protein levels of cleaved-caspase-3 (**A**,**B**) and phosphorylation levels of Bcl-2-associated death promoter (BAD) (**C**,**D**) in human granulosa cells. KGN (**A**,**C**) and hGC (**B**,**D**) cells were plated and incubated in various concentrations of GSE and GSPB2 (0, 1, 10, 50, and 100 μg/mL) for 24 h in appropriate medium with serum. Then, cells were lysed and proteins were separated by SDS-PAGE. The levels of Ser cleaved-caspase-3 and total caspase-3 (**A**,**B**), 136-phosphorylated BAD and BAD (**C**,**D**), were analyzed by probing with the appropriate antibodies as described in Materials and Methods. Representative blots in response to different concentrations of GSE are shown. The results are means ± SEM of four independent cultures with each condition in quadruplet. For hGC cells, an independent culture was from a pool of cells collected from three patients. * and *** represent a significant effect of GSE as compared to the control at *p* ≤ 0.05 and *p* ≤ 0.001, respectively. ° and °°° represent a significant effect of GSPB2 as compared to the control at *p* ≤ 0.05 and *p* ≤ 0.001, respectively.

Caspase-3 cleavage is a critical step in the control of the apoptotic DNA fragmentation [33]. We investigated whether this process was involved in the GSE and GSPB2 effect on apoptosis in human granulosa cells. As observed in Figure 4 and Supplemental Figures S7–S10, GSE (50 and 100 μg/mL) increased by 8- and 10-fold the amount of cleaved-caspase-3 (Figure 4A and Supplemental Figure S7 (KGN), *p* = 0.01 (50 μg/mL) and *p* < 0.001 (100 μg/mL); Figure 4B and Supplemental Figure S8 (hGC), p = 0.01 (50 μg/mL) and *p* < 0.001 (100 μg/mL) as compared to the respective control). Phosphorylation and inactivation of the pro-apoptotic Bcl-2 family member, Bcl-2-associated death promoter (BAD), are also involved in the apoptosis [34]. We examined the phosphorylation status of BAD by using an antibody recognizing phosphorylated Ser-136. We observed that the GSE treatment reduced by two- and four-fold BAD Ser-136 phosphorylation in KGN and hGC, respectively. (Figure 4C and Supplemental Figure S9 (KGN), *p* = 0.02 (50 μg/mL) and *p* < 0.001 (100 μg/mL); Figure 4D and Supplemental Figure S10 (hGC), *p* = 0.01 (50 μg/mL) and *p* < 0.001 (100 μg/mL) as compared to the respective control). Similar data were found in response to the GSBP2 treatment. These data indicate that GSE and GSBP2 treatments from 50 and 100 μg/mL were pro-apoptotic by increasing the caspase-3 cleavage and reducing the BAD phosphorylation.

### 2.5. Effect of GSE and GSPB2 Treatments on Oxidative Stress in Human Granulosa Cells 

**Figure 5:**
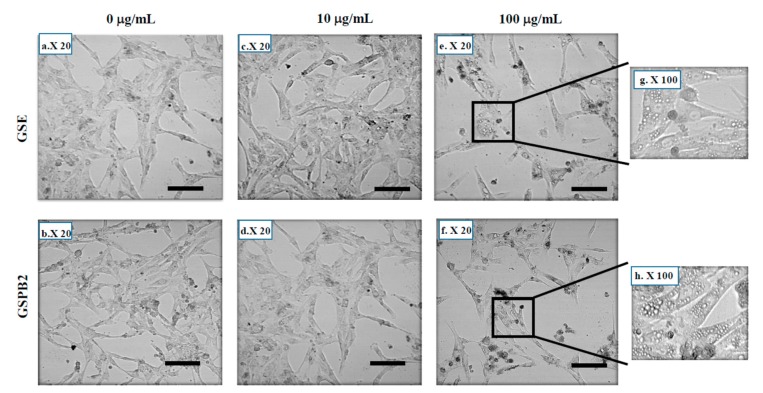
Morphological changes of KGN cells induced by the GSE and GSPB2 treatments for 24 h. KGN were cultured in medium with serum in the presence or in the absence of various doses of GSE or GSPB2 (0, 10, and 100 μg/mL). Photographs were taken 24 h after incubation with GSE and GSPB2. In the presence of 100 μg/mL of GSE or GSPB2, the KGN cells appeared smaller and wrinkled under the invert microscope and exhibited the classical morphological characteristics of cell death including cell atrophy and increased vacuoles. Scale bars = 25 μm (**a**–**f**). Black rectangles in (**e**,**f**) are magnified in (**g**,**h**), respectively.

As shown in Figure 5, we observed that the KGN cells treated with GSE and GSPB2 (100 μg/mL) displayed typical shrinkage and membrane bubbling as apoptosis characteristics. 

**Figure 6:**
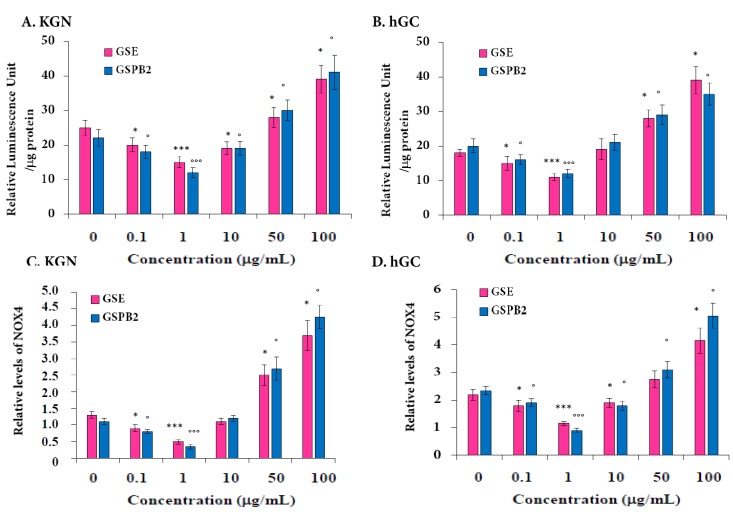
Determination of ROS content by using ROS-Glo assay (**A**,**B**) and mRNA expression of *NOX4* (**C**,**D**) in response to GSE and GSPB2 treatments in human granulosa cells. KGN (**A**,**C**) and hGC (**B**,**D**) cells were plated 24 h before the experiment in 24-well plates. After that, cells were treated with different concentrations of GSE or GSPB2 (0, 1, 10, 50, and 100 μg/mL) for 24 h in appropriate culture medium containing serum. Cellular oxidative stress was considered by measuring the ROS using ROS-Glo assay as described in Materials and Methods. (**C**,**D**) mRNA expression of NOX4 by RT-qPCR in KGN (**C**) and hGC (**D**) cells. The results are means ± SEM of four independent cultures with each condition in duplicate. For hGC cells, an independent culture was from a pool of cells collected from three patients. * and *** represent a significant effect of GSE as compared to the control at *p* ≤ 0.05 and *p* ≤ 0.001, respectively. ° and °°° represent a significant effect of GSPB2 as compared to the control at *p* ≤ 0.05 and *p* ≤ 0.001, respectively.

Several studies have demonstrated that apoptosis in various cell types, including granulosa cells, could be associated with an increase in reactive oxygen species (ROS) levels [12]. We determined the effect of GSE and GSPB2 treatments on the total ROS content. As shown in Figure 6A,B, GSE and GSPB2 treatments significantly decreased the amount of ROS in both KGN and hGC cells at the 0.1 and 1 μg/mL concentration (Figure 6A (KGN), GSE treatment *p* = 0.04 (0.1 μg/mL) and *p* < 0.001 (1 μg/mL), GSPB2 treatment *p* = 0.03 (0.1 μg/mL) and *p* < 0.001 (1 μg/mL); Figure 6B (hGC), GSE treatment *p* = 0.035 (0.1 μg/mL) and *p* < 0.001 (1 μg/mL), and GSPB2 treatment *p* = 0.03 (0.1 μg/mL) and *p* < 0.001 (1 μg/mL)). In contrast, with higher concentrations, 50 and 100 μg/mL, the GSE and GSPB2 treatments largely increased the ROS content in both KGN and hGC cells (Figure 6A (KGN), GSE treatment *p* = 0.04 (50 μg/mL) and *p* < 0.001 (100 μg/mL), and GSPB2 treatment *p* = 0.04 (50 μg/mL) and *p* < 0.001 (100 μg/mL) as compared to the respective controls; Figure 6B (hGC), GSE treatment *p* = 0.04 (50 μg/mL) and *p* < 0.001 (100 μg/mL), and GSPB2 treatment *p* = 0.035 (50 μg/mL) and *p* < 0.001 (100 μg/mL) as compared to the respective controls). To strengthen these data we investigated the effect of GSE and GSPB2 on the mRNA expression of the NADPH oxidases 4 (*NOX4*), one of main ROS production enzymes present in human granulosa cells [35]. As shown in Figure 6C,D, we found similar effects of GSE and GSPB2 on mRNA *NOX4* expression and ROS production in both KGN and hGC cells (Figure 6C (KGN), GSE treatment *p* = 0.03 (0.1 μg/mL), *p* = 0.04 (50 μg/mL), and *p* < 0.001 (1 and 100 μg/mL), and GSPB2 treatment *p* = 0.04 (0.1 and 50 μg/mL) and *p* < 0.001 (1 and 100 μg/mL) as compared to the respective controls; Figure 6D (hGC), GSE treatment *p* = 0.04 (0.1 and 50 μg/mL) and *p* < 0.001 (1 and 100 μg/mL), and GSPB2 treatment *p* = 0.03 (0.1 μg/mL), *p* = 0.04 (50 μg/mL), and *p* < 0.001 (1 and 100 μg/mL) as compared to the respective controls).

### 2.6. Effect of GSE and GSPB2 Treatments on Steroidogenesis in Human Granulosa Cells 

**Figure 7:**
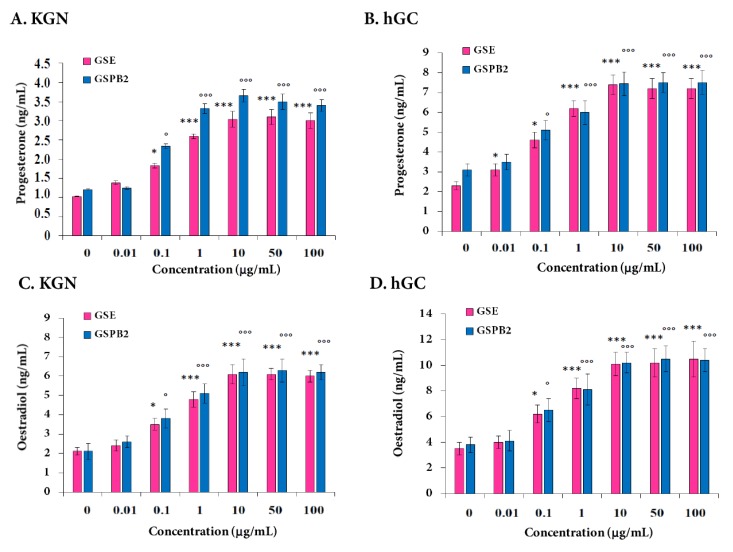
Effect of the GSE and GSPB2 treatments on progesterone (**A**,**B**) and estradiol (**C**,**D**) secretion by human granulosa cells. KGN (**A**,**C**) and hGC (**B**,**D**) cells were cultured for 48 h in medium with serum in the presence or in the absence of various doses of GSE and GSPB2. Progesterone (**A**,**B**) and estradiol (**C**,**D**) contents in the culture medium were then determined by ELISA. Results are expressed as ng/mL/100μg protein. Results are reported as means ± SEM of three independent experiments. * and *** represent a significant effect of GSE as compared to the control at *p* < 0.05 and *p* < 0.001, respectively. ° and °°° represent a significant effect of GSPB2 as compared to the control at *p* ≤ 0.05 and *p* ≤ 0.001, respectively.

We next investigated the effect of the GSE and GSPB2 treatments on the production of progesterone and estradiol by KGN and hGC. Cells were incubated with various concentrations of both GSE and GSPB2 (0, 0.01, 0.1, 1, 10, 50, and 100 μg/mL) for 48 h. Secretions of both progesterone (Figure 7A,B) and estradiol (Figure 7C,D) were increased by GSE and GSPB2 in a dose-dependent manner from 0.1 to 10 μg/mL and were unchanged from 10 to 100 μg/mL. These results were observed in both KGN and hGC cells (Figure 7A, GSE and GSBP2 treatments (0.1 μg/mL) *p* = 0.03 and *p* = 0.01, respectively, and *p* < 0.001 in both treatments at 1, 10, 50, and 100 μg/mL as compared to the respective controls; Figure 7B, both GSE and GSBP2 treatments (0.1 μg/mL) *p* = 0.01, and at 1, 10, 50, and 100 μg/mL *p* < 0.001 as compared to the respective controls; and the same p values were observed in Figure 7C,D as in Figure 7B).

**Figure 8:**
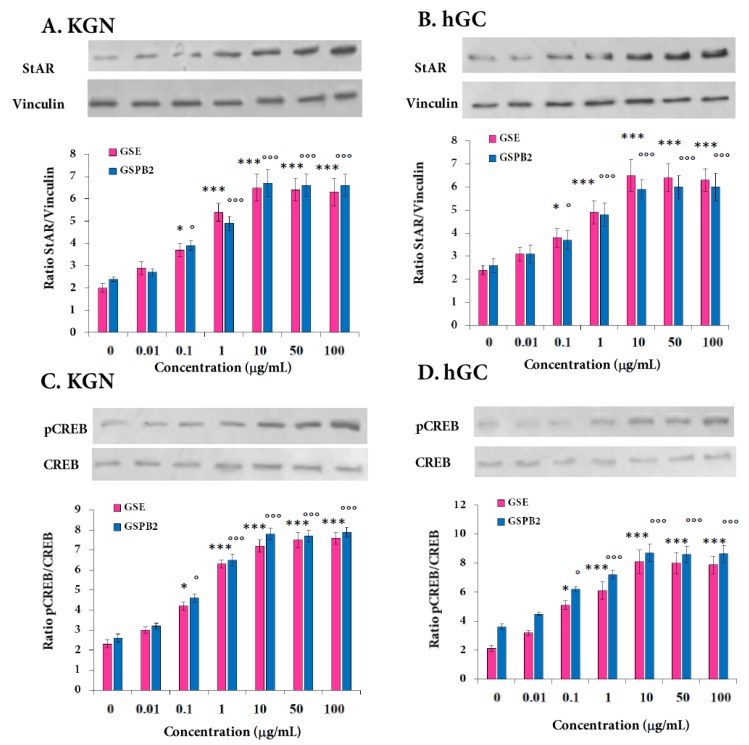
Effect of the GSE and GSPB2 treatments on the protein level of the cholesterol carrier StAR (**A**,**B**) and the phosphorylation level of transcription factor, CREB (**C**,**D**), in human granulosa cells. Protein extracts from KGN (**A**,**C**) and hGC (**B**,**D**) cells cultured for 48 h in the presence or absence of various concentrations of GSE and GSPB2 (0, 0.01, 0.1, 1, 10, 50, and 100 μg/mL) were subjected to SDS-PAGE, as described in Materials and Methods. The membranes were incubated with antibodies raised against the StAR (**A**,**B**), and phospho-CREB (**C**,**D**). Equal protein loading was checked by reprobing the membrane with an anti-vinculin and total CREB antibodies, respectively. Results are representative of at least three independent experiments. Blots were quantified, and the StAR/Vinculin and phospho-CREB/CREB ratios are shown. Representative blots in response to different concentrations of GSE are shown. The results are expressed as means ± SEM. * and *** represent a significant effect of GSE as compared to the control at *p* ≤ 0.05 and *p* ≤ 0.001, respectively. ° and °°° represent a significant effect of GSPB2 as compared to the control at *p* ≤ 0.05 and *p* ≤ 0.001, respectively.

We next determined whether the positive effect of GSE and GSPB2 treatments on steroid production was due to effects on StAR, an important cholesterol carrier. As shown in Figure 8A,B, GSE and GSPB2 treatments increased, in a dose-dependent manner, StAR protein levels from 0.1 to 10 μg/mL in both KGN and hGC cells (Figure 8A,B and Supplemental Figures S11 and S12, both GSE and GSBP2 treatments at 0.1 μg/mL *p* = 0.01 and *p* < 0.001 in both treatments at 1, 10, 50, and 100 μg/mL as compared to the respective controls). Furthermore, in both KGN and hGC, we demonstrated at a similar range of concentration, a significant increase in phosphorylation of cyclic AMP response element-binding protein (CREB) (Figure 8C,D and Supplemental Figures S13 and S14, both GSE and GSBP2 treatments at 0.1 μg/mL *p* = 0.01 and *p* < 0.001 in both treatments at 1, 10, 50, and 100 μg/mL as compared to the respective controls) which is a member of the transcription factor family involved in various cell functions, including steroidogenesis [36]. Thus, GSE and GSPB2 (0.1 to 10 μg/mL) improved steroidogenesis and these data were associated with an increase in phosphorylation of CREB and in StAR protein expression levels.

### 2.7. Effect of GSE and GSPB2 Treatments on Various Signaling Pathways in Human Granulosa Cells

**Figure 9:**
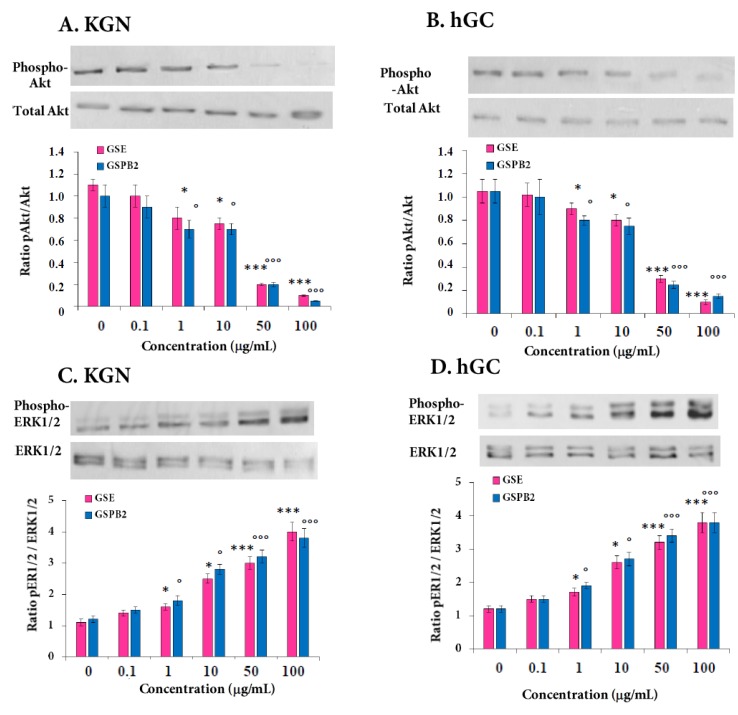
Dose effect of GSE and GSPB2 on the phosphorylation level of Akt (**A**,**B**) and MAPK ERK1/2 (**C**,**D**) in human granulosa cells. KGN (**A**,**C**) and hGC (**B**,**D**) cells were cultured for 10 min in medium with serum in the presence or in the absence of various doses of GSE or GSPB2 (0, 0.1, 1, 10, 50, and 100 μg/mL). Cells were then lysed and subjected to Western blotting with antibodies against phospho-Akt and Akt (**A**,**B**) and phospho-MAPK ERK1/2 (**C**,**D**). Representative blots in response to different concentrations of GSE are shown. Blots were quantified, and the phosphorylated protein/total protein ratio is shown. The results are presented as means ± SEM. * and *** represent a significant effect of GSE as compared to the control at *p* ≤ 0.05 and *p* ≤ 0.001, respectively. ° and °°° represent a significant effect of GSPB2 as compared to the control at *p* ≤ 0.05 and *p* ≤ 0.001, respectively.

In order to better understand the molecular mechanisms associated with the effects of GSE and GSPB2 in human granulosa cells, we analyzed MAPK ERK and Akt signaling pathways that are involved in many cell functions. As shown in Figure 9 and Supplemental Figures S15–S18, GSE and GSPB2 treatments (50 and 100 μg/mL) significantly inhibited phosphorylation of Akt (Figure 9A,B and Supplemental Figures S15 and S16, both GSE and GSBP2 treatments at 1 and 10 μg/mL *p* = 0.01 and *p* < 0.001 at 50 and 100 μg/mL in both treatments as compared to the respective controls) and increased MAPK ERK1/2 in a dose-dependent manner from 1 to 100 μg/mL (Figure 9C,D and Supplemental Figures S17 and S18, both GSE and GSBP2 treatments at 1 and 10 μg/mL *p* = 0.01 and *p* < 0.001 in both treatments at 50 and 100 μg/mL as compared to the respective controls) in both KGN and hGC cells. 

## 3. Discussion

**Figure 10:**
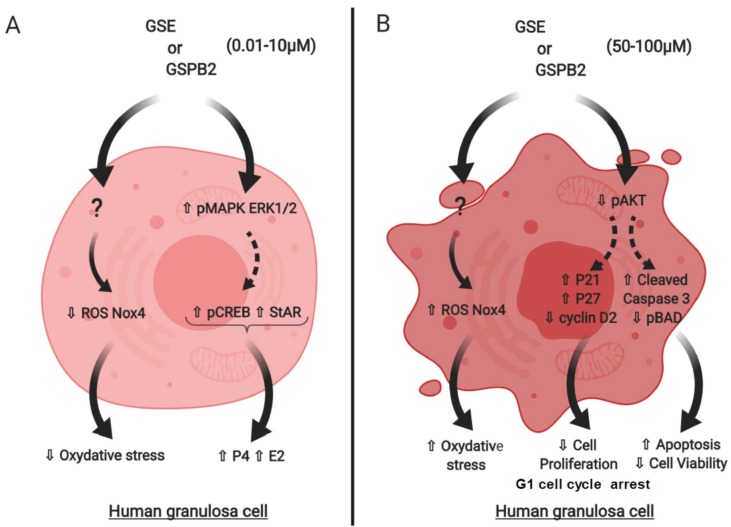
Schema representative of the effects of GSE and GSPB2 at different concentrations, (**A**) 0.01 to 10 μg/mL and (**B**) 50 to 100 μg/mL, on cell proliferation and viability, oxidative stress, steroidogenesis, and cell signaling in human granulosa cells. The question mark means that we don’t know the molecular mechanisms through GSE and GSPB2 bind to the cells. Dotted arrows suggest that an increase in MAPK ERK1/2 phosphorylation could explain an increase in CREB phosphorylation and StAR protein levels. Moreover, a decrease in Akt phosphorylation could induce an increase in p21, p27 and cleaved caspase 3 levels whereas it could decrease cyclin D2 protein and BAD phosphorylation levels.

In this study, we showed that GSE and GSPB2 treatments for 24 or 48 h modulate in vitro human granulosa cells function in a dose-dependent manner. From 0.1 to 10 μg /mL, GSE and GSPB2 treatments decreased the ROS cell content and NOX4 mRNA expression without affecting cell proliferation and viability. In contrast, at higher concentrations (50 to 100 μg/mL), GSE and GSPB2 impaired cell proliferation and induced a G1 cell cycle arrest. These data were associated with a decrease in cyclin D2 protein expression and an increase in the CDKI, and p21 and 27 mRNA expression. Moreover, in these latter conditions, GSE and GSPB2 induced cell death, an increase in the cleaved-caspase-3 and BAD phosphorylation levels, and a strong inhibition in Akt phosphorylation level (Figure 10). These negative effects were also associated with a higher level of oxidative stress and morphology changes suggesting that GSE or GSPB2 at high concentrations could exert some deleterious effects in ovarian cells. Interestingly, the GSE and GSPB2 treatments (0.1 to 100 μg/mL) improved steroidogenesis and this was associated with an increase in StAR protein expression, CREB, and MAPK ERK1/2 phosphorylation.

### 3.1. GSE and Cell Proliferation and Death

In our experiments, we observed that GSE and GSBP2 treatments with a concentration lower than 10 μg/mL in the culture medium did not affect the growth of human granulosa cells. However, from a concentration of 50 μg/mL, they inhibited proliferation and viability in both KGN and hGC cells. This negative effect with similar concentrations has been described in various human tumor cell lines (human oesophageal squamous cancer cell line ECA109, [37,38] and primary cells [39]). In our experiment, we observed that these negative effects of GSE and GSBP2 were associated with a reduction of cyclin D2 expression and BAD phosphorylation and also with an increase in CDKIs p21 and p27 and cleaved-caspase-3 levels. In parallel, we showed a strong inhibition of Akt phosphorylation. Akt is a serine and threonine protein kinase, also known as protein kinase B, that regulates a variety of cellular processes including survival, proliferation, protein translation, and metabolism [40]. In many cell types, including ovarian cells, Akt activation is involved in the cell cycle progression by regulating cyclin D’s function [41]. In our present work, we studied only cyclin D2 since it is mainly expressed and regulated in granulosa cells [42]. The Akt signaling pathway has been shown to control cell cycle arrest at phases G1 and S by regulating *cyclin D2* gene transcription in rat granulosa cells but also by modulating p27 and p21 CDKI transcriptionally and post-translationally [43]. BAD, the Bcl-2-associated death promoter protein, is a pro-apoptotic member of the *Bcl-2* gene family which is involved in initiating apoptosis [44]. The BAD function is modulated by phosphorylation at two sites, serine 112 (Ser-112) and serine 136 (Ser-136). In the absence of phosphorylation of these sites, BAD is thought to induce cell death, possibly via the formation of heterodimers with BCL-XL at the mitochondrial membrane and a decrease of its binding to 14-3-3 protein in the cytosol [45], as well as the concomitant generation of BAX homodimers (pro-apoptotic factors). It has been speculated that Akt inhibits apoptosis by maintaining the Bcl-x function and preventing cytochrome c release from mitochondria. Akt phosphorylates BAD at Ser-136 in vitro and in vivo. It is also well known that Akt phosphorylates caspase-9 and regulates caspase-3 cleavage level which is a critical step in the control of the apoptotic DNA fragmentation [33]. Thus, an inhibition of Akt phosphorylation in response to GSE and GSBP2 (50 and 100 μg/mL) could inhibit cyclin D2 expression through an increase in p21 and p27 CDKI to induce a G1 cell cycle arrest and reduce cell proliferation in human granulosa cells. It can also impair BAD phosphorylation and increase cleaved-caspase-3 to induce death. Agarwal et al. showed that a polyphenolic fraction from grape seeds causes growth inhibition of breast carcinoma MDA-MB468 and DU145 human prostate carcinoma cells by inhibiting mitogen-activated protein kinases activation and inducing G1 arrest [46]. In our experiment, GSE or GSBP2 did not impair MAPK ERK1/2 phosphorylation. To explain this discrepancy, we hypothesize that the phytochemical profile of grape seed extract was different in the studies or the GSE could use different signaling pathways to reduce cell proliferation according to the cell type.

### 3.2. GSE and Cell Steroidogenesis

In our study, the GSE and GSBP2 treatments in human granulosa cells significantly increased the secretion of progesterone and estradiol and the protein levels of StAR which is involved in the transport of cholesterol from the outer to the inner mitochondrial membrane [47]. We showed that GSE and GSBP2 increased in a dose-dependent manner MAPK ERK1/2 and CREB (cAMP response element binding protein) phosphorylation. Several studies showed that MAPK ERK1/2 can activate CREB [48] that binds as a dimer to a sequence found in the regulatory region of cAMP-regulated genes, including StAR [49]. Thus, these data suggest that the GSE and GSBP2 treatments could increase steroidogenesis through MAPK ERK1/2 and then CREB phosphorylation that could increase StAR expression in human granulosa cells. In 2003, Eng reported on the ability of grape seed extract (GSE) to act as an aromatase inhibitor in vitro as well as in vivo in aromatase-transfected MCF-7 (MCF-7aro) BC xenograft mice [50]. However, most of the study used breast cancer cell lines and in our study, we observed a positive effect on steroid production in granulosa cells suggesting a cell specific effect. 

### 3.3. GSE Oxidative Stress

It is well known that polyphenolic compounds have antioxidant properties. They are potent-free radial scavengers. Oxidative stress is an important inducement in ovarian aging which results in fecundity decline in humans and different animals. In our study, we showed that GSE and GSBP2 treatments at 0.1 to 1 μg/mL reduced the ROS content and *NOX4* mRNA expression, whereas, the opposite effects were observed at 50–100 μg/mL in human granulosa cells. The only function of the enzyme family NADPH oxidases (NOXs) is the generation of reactive oxygen species (ROS) and mainly H_2_O_2_. Since NOX4 is the main NOX enzyme expressed in granulosa cells [35], we studied the expression of this enzyme. From our study, we can say that GSE and GSBP2 are able to regulate the ROS content in human granulosa cells, probably through a transcriptional effect on NOX4. Several data observed that GSE may have negative or beneficial effects through modulation of NOX actions [51]. Concerning the impact of GSE on fertility, it would be interesting to evaluate their effects on the oocyte maturation and embryo development. One study performed in sheep showed beneficial effects on oocyte maturation and early development based on the mean numbers of cleavage, morula, and blastocyst rates. However, this effect was observed with a high GSE concentration (800 µg/mL) [52]. Moreover, grape seed proanthocyanidin extracts have been demonstrated to reduce the cadmium-induced meiosis inhibition during oogenesis in chicken embryos [53].

### 3.4. Mode of Action of GSE in Human Granulosa Cells 

It could be interesting to determine which molecular mechanism of GSE and GSBP2 can mediate their action through the plasma membrane of granulosa cells. In intestinal cells (Caco-2 cells), grape seed procyanidin extract is a co-agonist ligand for the farnesoid X receptor (FXR), which is a nuclear hormone receptor involved in the regulation of lipid and glucose metabolism [54]. In insulin sensitive cells, it has been demonstrated that grape seed procyanidin extracts (GSPE) are able to bind to the insulin receptor (IR) and induce IR autophosphorylation on tyrosine residues, suggesting another molecular mechanism for GSE and GSBP2 [55]. In granulosa cells, IR and FXR signaling are present and regulate steroidogenesis and cell growth [56], and therefore, in this study, GSE and GSBP2 could mediate their effects through IR or FXR signaling. 

### 3.5. Limitations and Perspectives

Although this study provided meaningful data, it has some limitations. Firstly, we performed in vitro experiments in both human primary granulosa cells and the KGN cells line and, consequently, we cannot predict the in vivo effect of GSE and GSBP2 on ovarian functions. Indeed, we still do not know which procyanidins or metabolites of procyandins will intercact in vivo with granulosa cells. Furthermore, we analyzed the effects of GSE and GSBP2 in non-pathological human primary granulosa cells and, next, we plan to investigate the effect of these extracts on granulosa cells from obese or PCOS (Polycystic Ovary Syndrome) patients. Indeed, obesity or insulin resistance may increase the oxidative stress of these cells and it would be interesting to show that grape seed extracts are potent antioxidants molecules. 

## 4. Materials and Methods

### 4.1. Patients

The present study was performed according to the rules set in the Declaration of Helsinki. Patients gave their written informed consent and did not receive any monetary compensation for participating in the study. The experiment was approved by the Institutional Review Board (Authorization protocol number 2016_075, date of approval: 1 January 2016, Ethics Committee of University Hospital of Tours, France). Patients undergoing an in vitro fertilization protocol were recruited in 2017 and 2018. The cause of infertility was mechanical, unexplained, or male factor infertility without any known endocrinopathy (PCOS, hyperprolactinemia, hypothyroidism, and hyperthyroidism). 

### 4.2. Isolation and Culture of Human Granulosa Cells

After isolation of cumulus–oocyte complexes, follicular fluid was recovered and centrifuged at 400 × g for 10 min to pellet cell remnants. Human granulosa cells from this pellet were then isolated, purified, and cultured as previously described [57].

### 4.3. Human Granulosa-Like Tumor Cell Line, KGN

The human ovarian granulosa-like tumor cell line, KGN, was obtained from Masatoshi Nomura and Hajime Nawata, Kyushu University, Japan [58]. The culture medium used for these cells was DMEM/ Ham’s F12 (Sigma, St. Louis MO) supplemented with 10% FCS and antibiotics (100 IU/ml penicillin and 100 μg/mL streptomycin obtained from Sigma). Cells were cultured in a 5% CO2 atmosphere at 37 °C.

### 4.4. Source of GSE and GSPB2 and Antibodies

GSE was donated by the INDENA Company (Milan, Italy) and used throughout the study. The GSE contains approximately 93.8% proanthocyanidins, with monomers (7.6%) dimers (8.3%), and oligomers (77.9%). Proanthocyanidin B2 (GSPB2) was obtained from Sigma (St. Louis MO). Antibodies to phospho-ERK1/2 (Thr202/Tyr204), AKT, phospho-BAD (phospho-Ser-136), BAD, cleaved-caspase-3, and total caspase-3 were purchased from New England Biolabs Inc. (Beverly, MA, USA) and antibodies to ERK2 (C14), cyclin D2 (C17), p21, p27, and phospho-AKT (Ser 473) were obtained from Santa Cruz Biotechnology (Santa Cruz, CA, USA). Monoclonal antibodies to vinculin were purchased from Sigma. Rabbit polyclonal antibodies against StAR were generously provided by Dale Buchanan Hales (University of Illinois, Chicago, IL, USA). All antibodies were used at 1/1000 dilution in Western blotting.

### 4.5. Western Blot

The KGN and hGC cells were solubilized in a lysis buffer for 30 min at 4 °C and centrifuged, as previously described [59]. Then, the supernatants were collected and the protein concentration was determined by using the BiCinchoninic Acid Assay (Interchim, Montluçon, France). Protein lysates (50 μg) were subjected to electrophoresis and then immunoblot was performed, as previously described [59]. Proteins were revealed by using enhanced chemiluminescence (Western Lightning Plus-ECL, Perkin Elmer, Villebon-sur-Yvette, France) a G-box SynGene (Ozyme, St Quentin en Yvelines, France), and a GeneSnap software (Ozyme, St Quentin en Yvelines, France), as previously described [59]. Then, proteins were analyzed with GeneTools software. The data are represented as the intensity signal in arbitrary units after normalization.

### 4.6. Thymidine Incorporation Assay

The cells were cultured in DMEM–Ham's F12 (KGN) or McCoy’s 5A (hGC) medium and 10% FBS, as described above, for 24 h. They were then overnight serum deprived followed by the addition of 1 µCi/μL of [3H]-thymidine (Becton Dickinson, Le Pont de Claix, France) in the presence or absence of different concentrations of GSE and GSPB2. Following 24 h of incubation, excess thymidine was removed after two washes with phosphate-buffered saline (PBS 1X) and cells were fixed with cold trichloacetic acid 50% for 15 min and lysed by 0.5 M NaOH. The amount of radioactivity in the cells was measured by scintillation fluid (Perkin Elmer, Courtaboeuf, France) counting in a β-photomultiplier.

### 4.7. Cell Viability

The cells (KGN and hGC) were incubated in various concentrations of GSE and GSPB2 (0, 0.01, 0.1, 1, 10, 50, and 100 μg/mL for 24 h in culture medium with serum. Then, cell viability was determined by using the Cell Counting Kit-8 (CCK8 reagent) assay or by trypan blue staining (Sigma, Saint Quentin Fallavier, France) following the manufacturer’s protocol. 

### 4.8. Flow Cytometry and Cell Cycle Analysis

After incubation with different GSE and GSPB2 concentrations (0, 1, 10, 50, and 100 μg/mL) for 24 h, the KGN cells were trypsinized, pelleted, and washed twice in PBS. The cells were resuspended in a citrate buffer and then the nuclei were stained as previously described [60]. Data were analyzed by flow cytometry on a FACSCalibur using CellQuest software. Cell cycle analysis was performed using ModfitLT™ software Version 2 (Verify Software House, Inc, Topsham, USA).

### 4.9. Progesterone (P4) and Estradiol (E2) Radioimmunoassay

The concentration of P4 and E2 in the culture medium of both KGN and hGC cells cultured for 48 h with GSE or GSPB2 was determined by using a radioimmunoassay protocol, as previously described [59]. Data are shown as means ± standard error of the mean (SEM) of three cultures of KGN and hGC. In each culture, each condition was analyzed for at least five separate wells.

### 4.10. Measuring Oxidative Stress in Response to GSE and GSPB2 by ROS-Glo Assay

The KGN and hGC cells were exposed to different doses of GSE and GSPB2 (0, 0.1, 1, 10, 50, and 100 μg/mL) for 24 h in culture medium with 10% FBS. Cellular oxidative stress was considered by measuring the ROS using the ROS-Glo H_2_O_2_ Assay kit (Promega, Charbonières les Bains, France) following the manufacturer’s protocol. 

### 4.11. RNA Extraction and RTqPCR

The total RNA from human granulosa cells incubated with or without different concentrations of GSE or GSPB2 was extracted by using the TRIzol® reagent (Sigma, Saint Quentin Fallavier, France) and then purified using a DNA-free kit™ according to the manufacturer’s recommendations (Invitrogen™ by Life Technologies™, Villebon sur Yvette, France). Generation of cDNAs by reverse transcription, real-time PCR, and analysis of the relative expression of the gene of interest were performed, as previously described [57].The *NOX4* primers used in our study were: forward, 5’CTT TTG GAA GTC CAT TTG AG3’ and reverse, 5’ATC AAG CGG CCC CCT TTT TTC AC3’; accession number: NM 016931 (Invitrogen™ by Life Technologies ™). The mRNA expression levels were standardized using the geometric mean of three reference genes (*ACTB*, *PPIA*, and *GAPDH*). The primers of the reference genes were: *ACTB* (forward, 5’-AAAGACCTGTACGCCAACAC-3’ and reverse, 5’-CTCAGGAGGAGCAATGATCTTG-3’; accession number: NM 001101), *PPIA* (forward, 5’-GCATACAGGTCCTGGCATCT-3’ and reverse, 5’-TGTCCACAGTCAGCAATGGT-3’; accession number: XM 024446809.1) and *GAPDH* (forward, 5’-TCAGCCGCATCTTCTTTTGC-3’ and reverse, 5’-ACGACCAAATCCGTTGACTC-3’; accession number: NM 002046). The use of the geometric mean of several reference genes has already been validated [61]. 

### 4.12. Statistical Analysis

All data are reported as means ± standard error of mean (SEM). Using the software package Excel and Statview 5.05 (SAS Campus Drive, Cary, NC, USA), we performed one *t*-test or one-way analysis of variance (ANOVA) to compare the different means. When the ANOVA indicated significant effects, the means were analyzed by using the Fisher's test.

## 5. Conclusions

Our in vitro data demonstrated that GSE and GSPB2 (0.1–10 μg/mL) treatments increase steroidogenesis and decrease oxidative stress without affecting cell proliferation and viability in KGN and primary human granulosa cells. Further experiments need to be performed to determine the molecular mechanism of GSE and GSPB2 on human granulosa cells and to validate the in vivo potential use of these natural phytoproducts to improve the quality of the ovarian cells and, consequently, fertility.

## Figures and Tables

**Table 1:** DNA content analysis by FACS in KGN cells in response to various concentrations of GSE and GSBP2 (*n* = 3 independent experiments). The mean of percentage of cells in each cell cycle stage (G0–G1, S, and G2–M) ± SEM is shown. In response to each treatment (GSE and GSBP2), the p value is indicated as compared to the control (no GSE or GSPB2 treatment) in each stage of cell cycle.

Cells in Cell Cycle							
Treatment (24 h)	Concentration (μg/mL)	G0–G1 (%)	*p* value	S (%)	*p* value	G2–M (%)	*p* value
**GSE**	0	54.9 ± 0.6	-*	31.6 ± 0.3	-	13.6 ± 0.9	-
	1	56.3 ± 1.4	0.61	29.7 ± 1.0	0.22	13.9 ± 0.4	0.75
	10	57.4 ± 1.0	0.08	29.7 ± 0.3	0.15	12.3 ± 0.9	0.62
	50	76.1 ± 2.0	0.02	19.4 ± 1.9	0.004	4.5 ± 0.3	0.003
	100	84.3 ± 0.6	0.001	11.9 ± 0.2	0.001	3.9 ± 0.4	0.001
**GSBP2**	0	52.3 ± 0.8	-	32.4 ± 0.4	-	15.2 ± 0.8	-
	1	53.3 ± 1.5	0.65	32.1 ± 1.1	0.72	14.5 ± 0.6	0.68
	10	54.7 ± 0.9	0.09	31.7 ± 0.5	0.61	13.7 ± 1.2	0.31
	50	75.2 ± 1.0	0.001	18.5 ± 1.4	0.004	6.3 ± 0.4	0.008
	100	86.4 ± 1.1	0.001	10.6 ± 0.5	0.001	3.1 ± 0.5	0.001

Note: * - = no *p* value.

## Supplementary Materials

Supplementary materials can be found at https://www.mdpi.com/1422-0067/20/17/4215/s1.
