# Supplementary material for: Effects of Grape Seed Extract and Proanthocyanidin B2 on In Vitro Proliferation, Viability, Steroidogenesis, Oxidative Stress, and Cell Signaling in Human Granulosa Cells
Source: Int J Mol Sci. 2019 Aug 28;20(17):4215. doi: 10.3390/ijms20174215 (PMC6747392; doi:10.3390/ijms20174215)
Supplement: Supplementary file 1 [file ijms-20-04215-s001.pdf]

Cyclin D2 (33 kDa) KGN cells

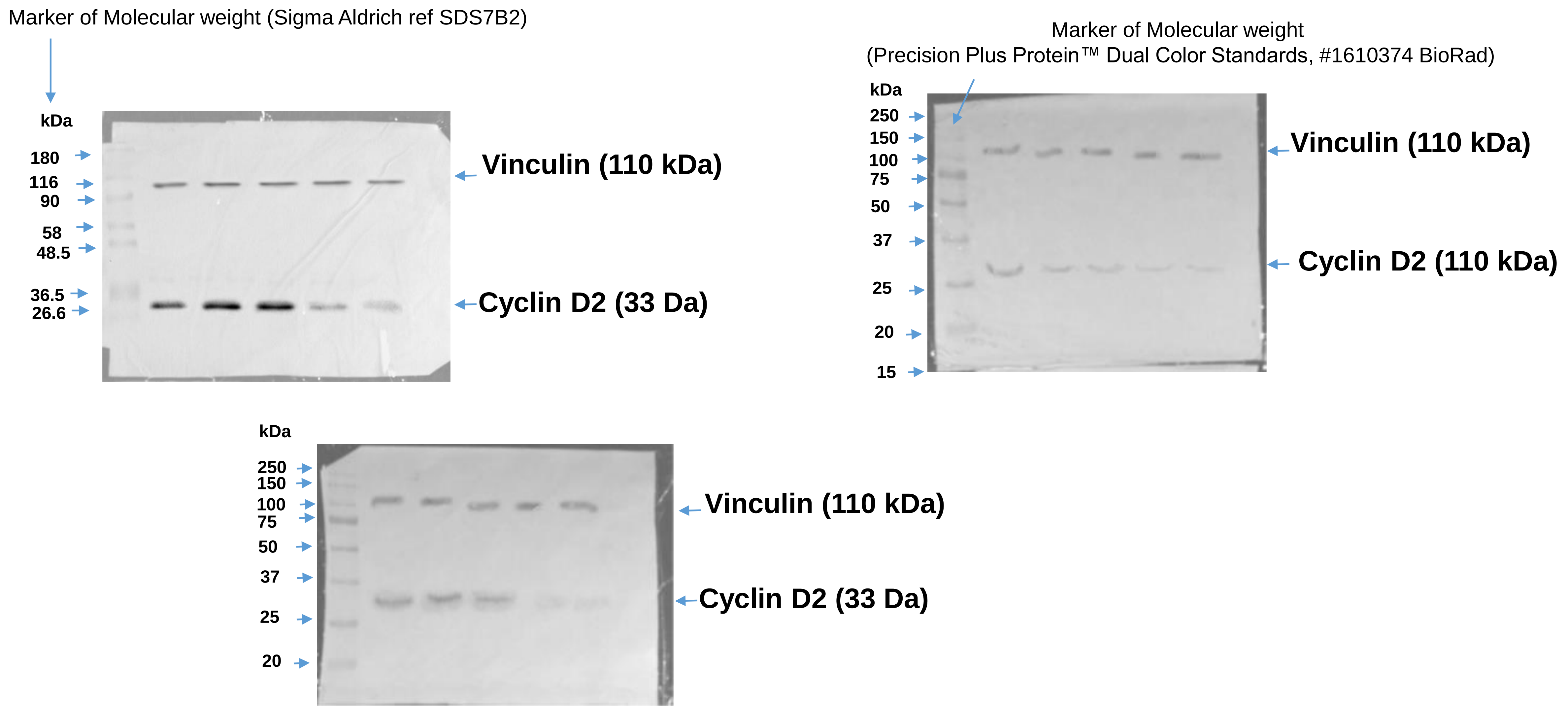

Revelation of the blots performed with the a G-box SynGene (Ozyme, St Quentin en Yvelines, France) and GeneSnap software : a composite with the membrane with the markers and the membrane revealed with ECL after the antibodies incubation is shown**.** Membranes were probed with cyclin D2 and Vinculin antibodies.

Supplemental figure 1

Cyclin D2 (33 kDa) hGC cells

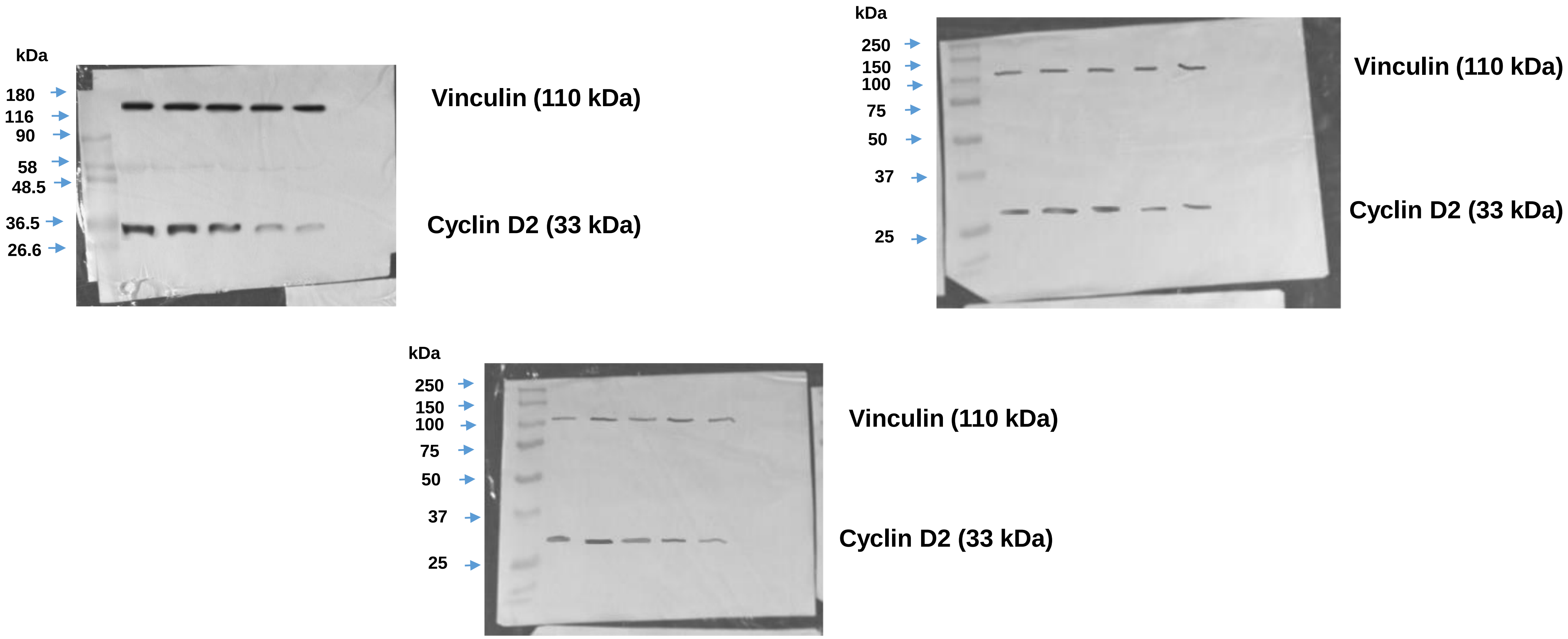

Revelation of the blots performed with the a G-box SynGene (Ozyme, St Quentin en Yvelines, France) and GeneSnap software : a composite with the membrane with the markers and the membrane revealed with ECL after the antibodies incubation is shown**.** Membranes were probed with cyclin D2 and Vinculin antibodies.

Supplemental figure 2

P21 (21 kDa) KGN cells

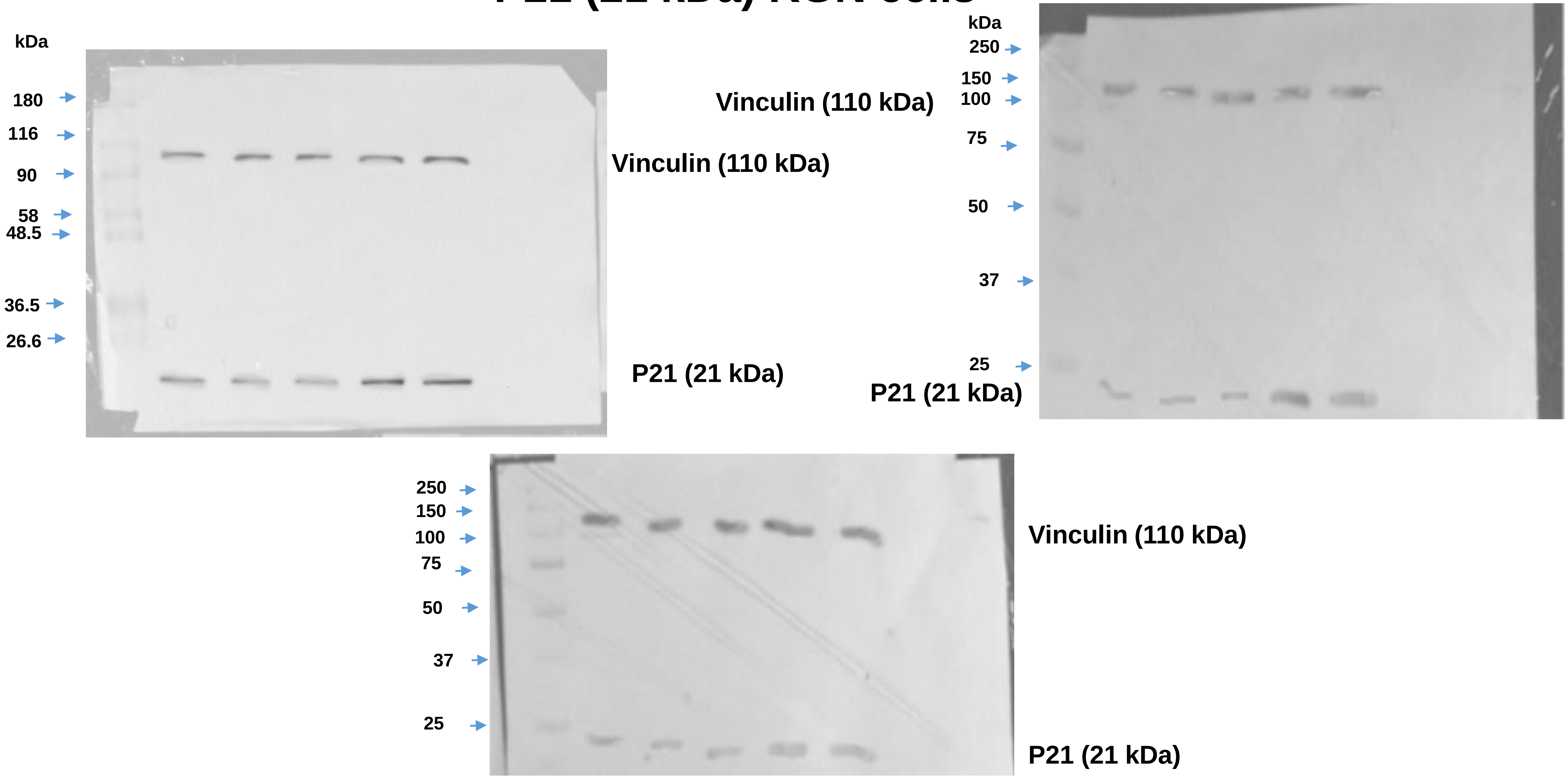

Revelation of the blots performed with the a G-box SynGene (Ozyme, St Quentin en Yvelines, France) and GeneSnap software : a composite with the membrane with the markers and the membrane revealed with ECL after the antibodies incubation is shown**.** Membranes were probed with p21 and Vinculin antibodies.

Supplemental figure 3

P21 (21 kDa) hGC cells

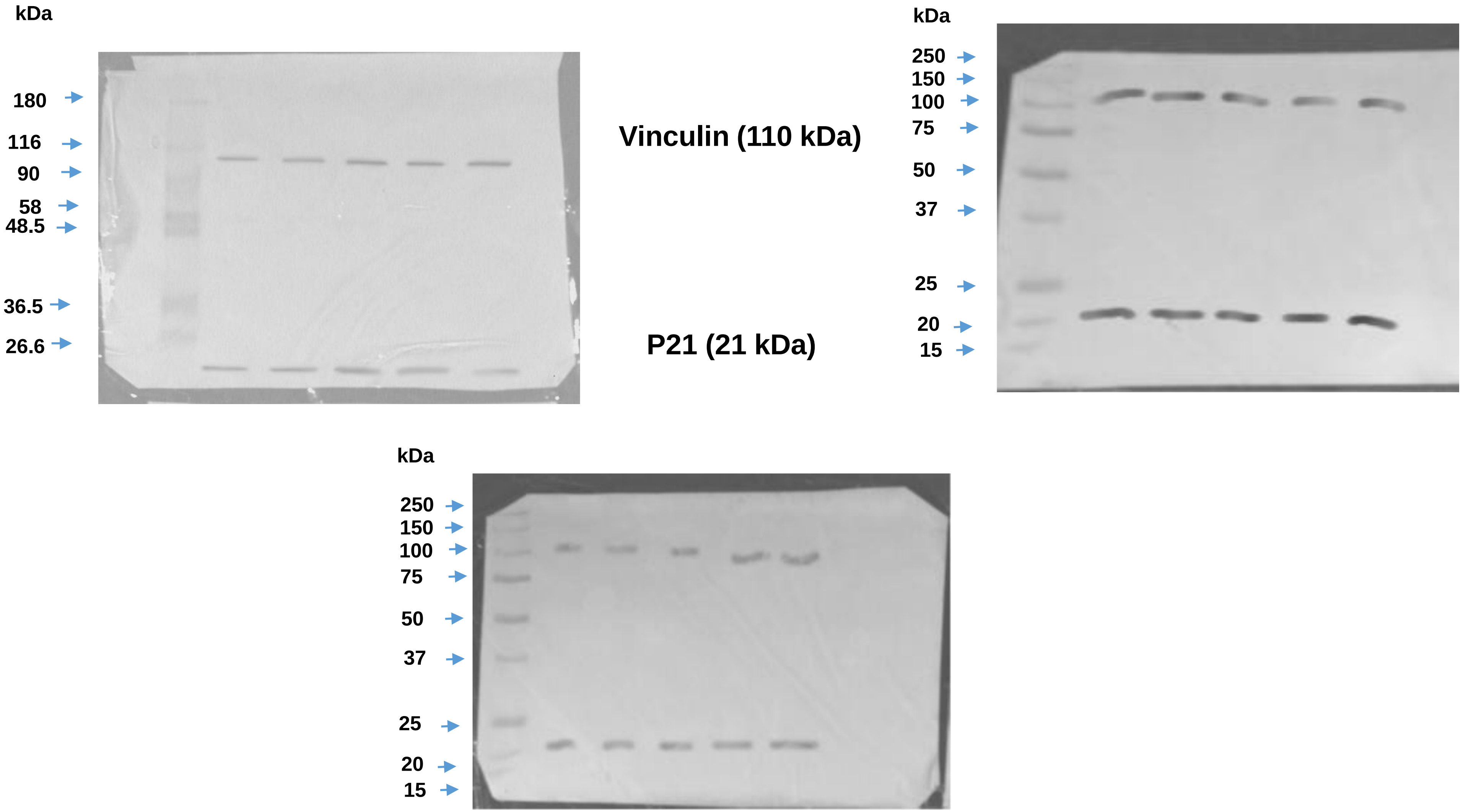

Revelation of the blots performed with the a G-box SynGene (Ozyme, St Quentin en Yvelines, France) and GeneSnap software : a composite with the membrane with the markers and the membrane revealed with ECL after the antibodies incubation is shown**.** Membranes were probed with p21 and Vinculin antibodies.

Supplemental figure 4

P27 (27 kDa) KGN cells

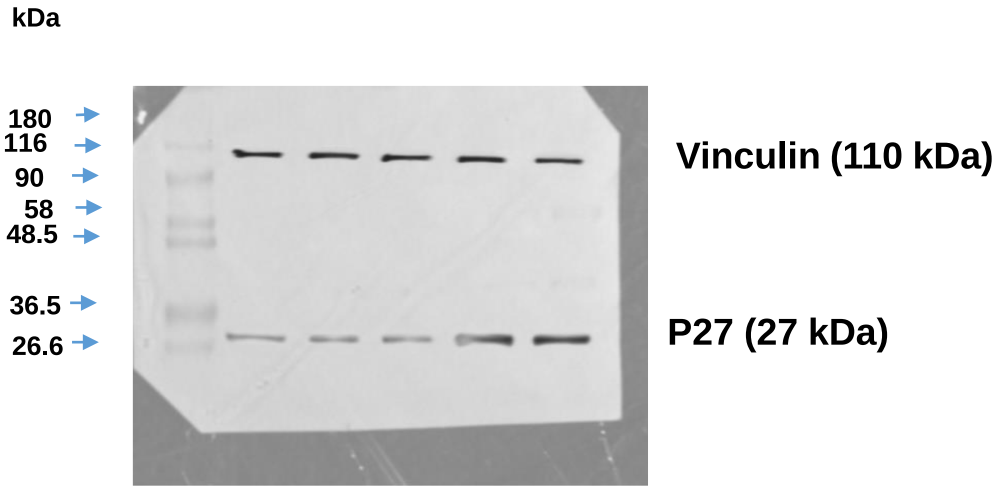

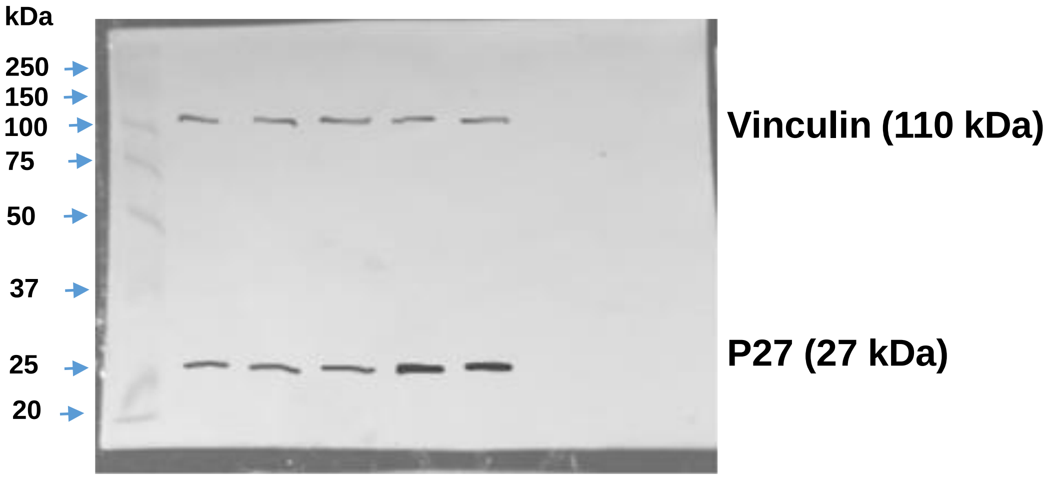

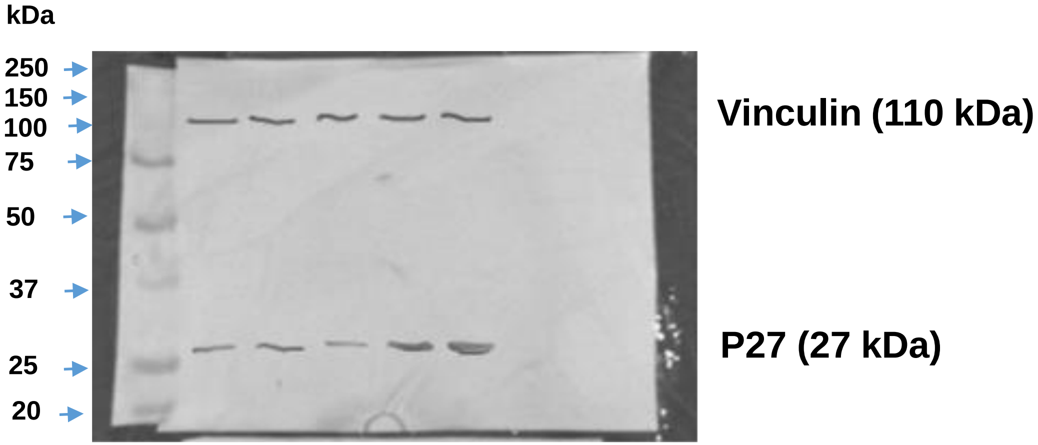

Revelation of the blots performed with the a G-box SynGene (Ozyme, St Quentin en Yvelines, France) and GeneSnap software : a composite with the membrane with the markers and the membrane revealed with ECL after the antibodies incubation is shown**.** Membranes were probed with p27 and Vinculin antibodies.

Supplemental figure 5

P27 (27 kDa) hGC cells

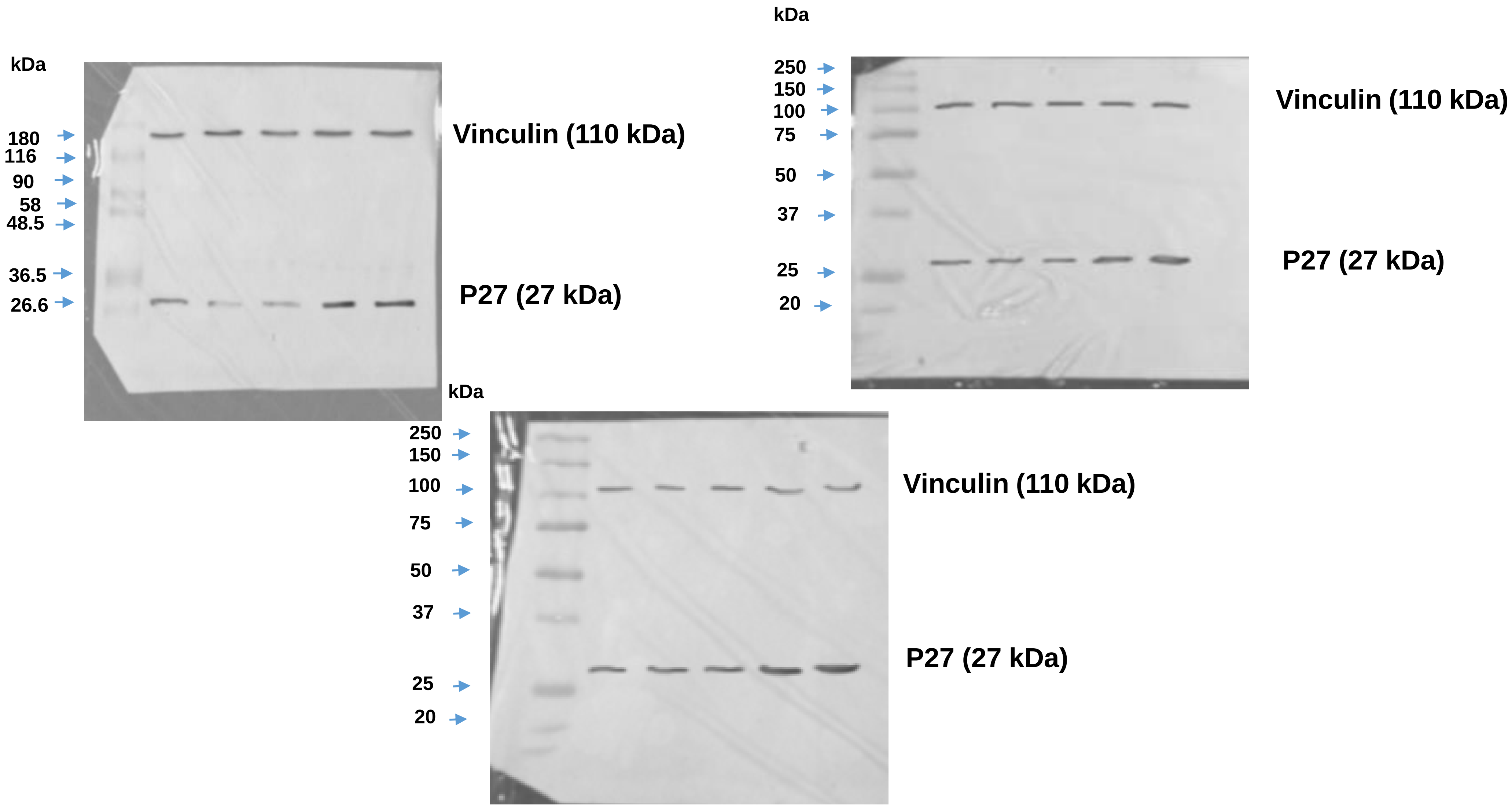

Gel SDS PAGE 12%, revelation of the blots performed with the a G-box SynGene (Ozyme, St Quentin en Yvelines, France) and GeneSnap software : a composite with the membrane with the markers and the membrane revealed with ECL after the antibodies incubation is shown**.** Membranes were probed with p27 and Vinculin antibodies.

Supplemental figure 6

Caspase 3 cleaved (18 kDa) in KGN cells

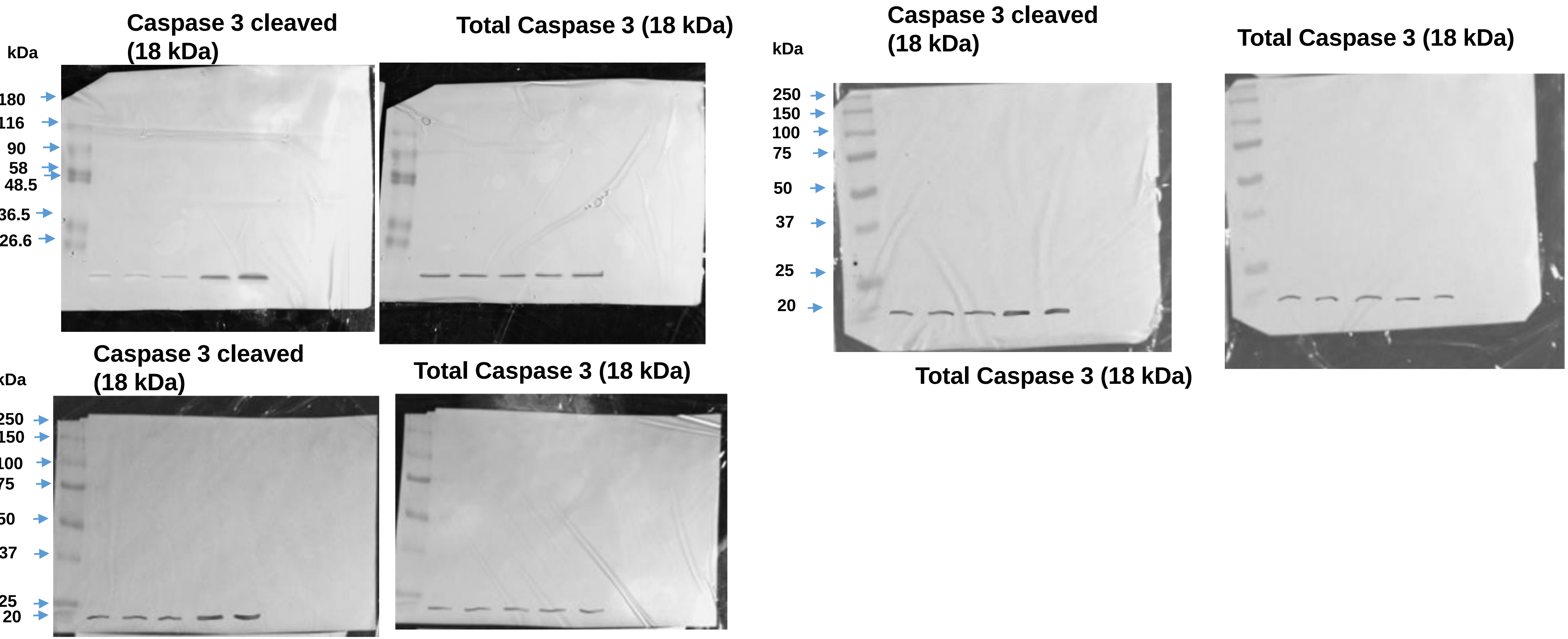

Gel SDS PAGE 12%, revelation of the blots performed with the a G-box SynGene (Ozyme, St Quentin en Yvelines, France) and GeneSnap software : a composite with the membrane with the markers and the membrane revealed with ECL after the antibodies incubation is shown**.** Membranes were probed with caspase 3 cleaved antibodies and then after stripping they were probed with with total caspase 3 antibodies.

Supplemental figure 7

Caspase 3 cleaved (18 kDa) in hGC cells

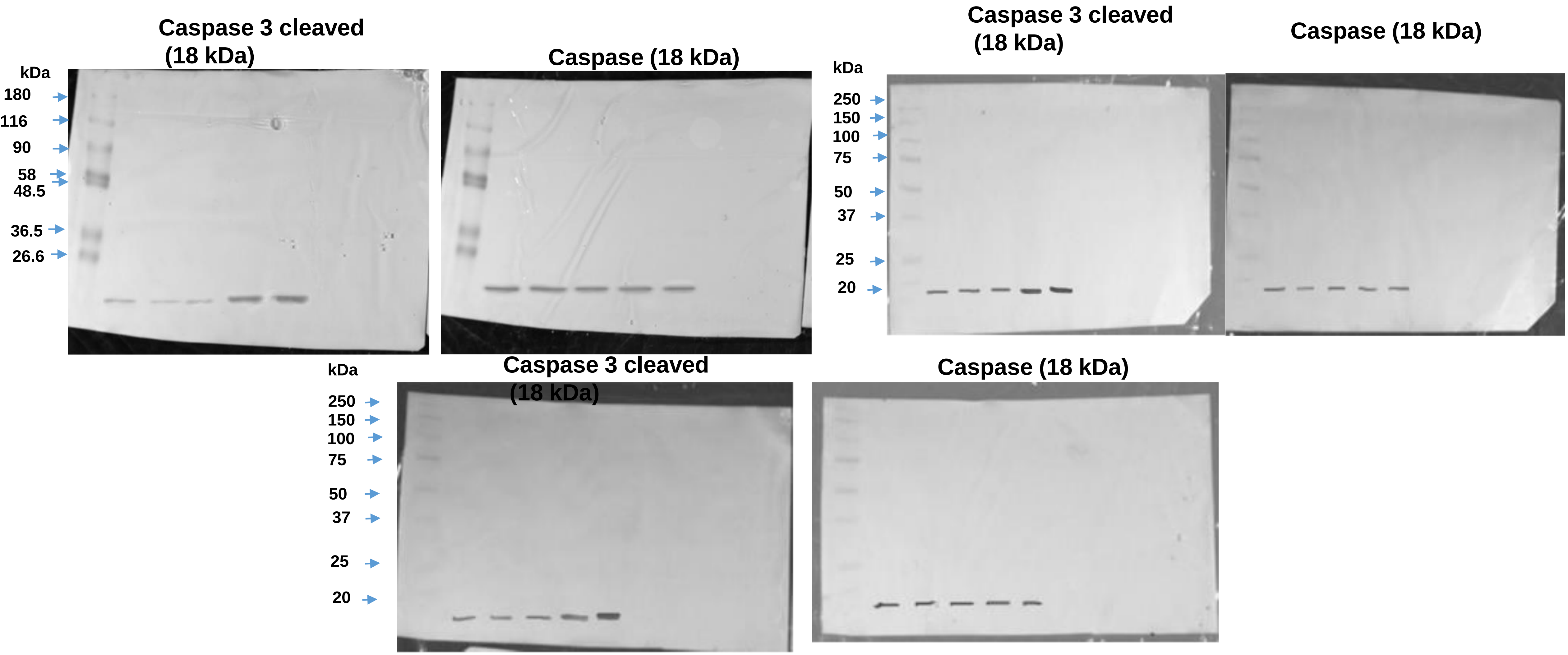

Revelation of the blots performed with the a G-box SynGene (Ozyme, St Quentin en Yvelines, France) and GeneSnap software : a composite with the membrane with the markers and the membrane revealed with ECL after the antibodies incubation is shown**.** Membranes were probed with caspase 3 cleaved antibodies and then after stripping they were probed with with total caspase 3 antibodies.

Supplemental figure 8

Phospho-Bad (23 kDa) in KGN cells

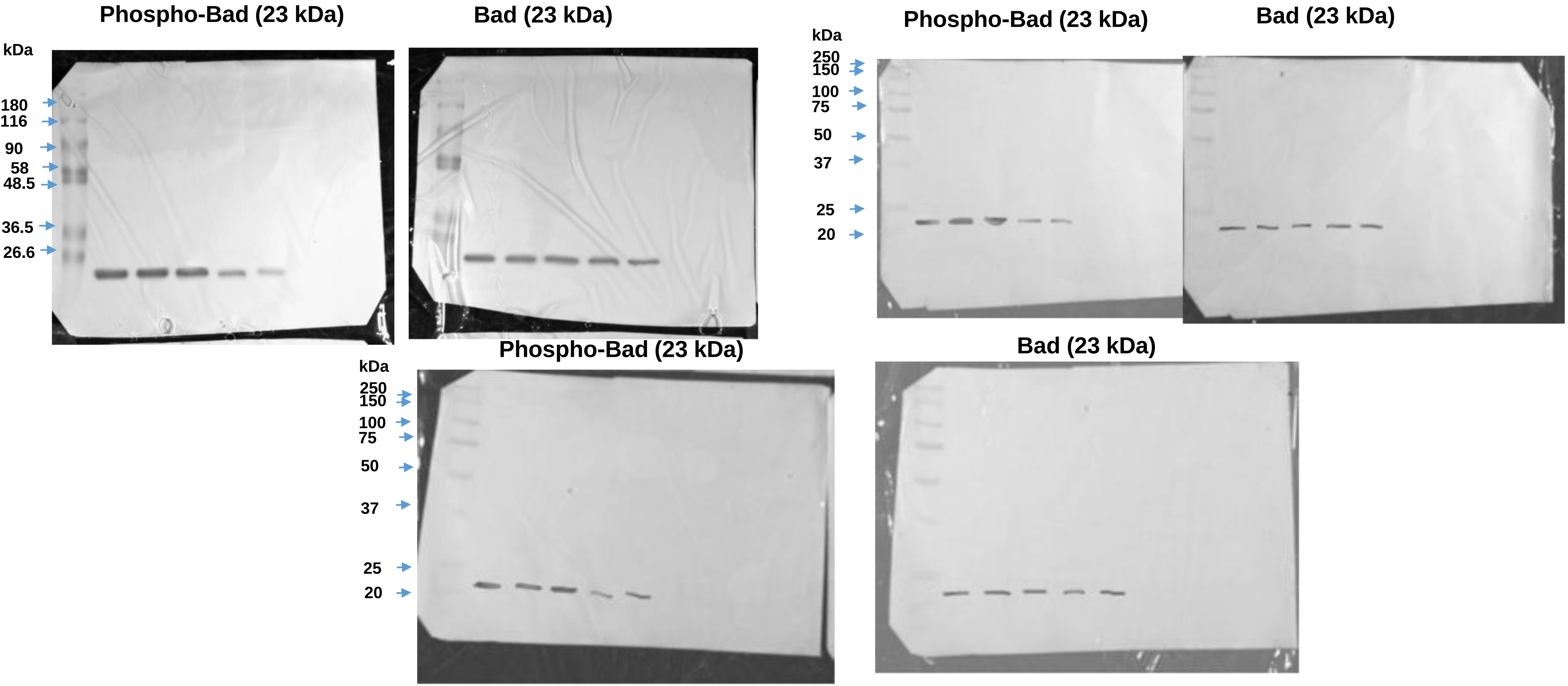

Revelation of the blots performed with the a G-box SynGene (Ozyme, St Quentin en Yvelines, France) and GeneSnap software : a composite with the membrane with the markers and the membrane revealed with ECL after the antibodies incubation is shown**.** Membranes were probed with phospho-Bad antibodies and then after stripping they were probed with total Bad antibodies.

Supplemental figure 9

Phospho-Bad (23 kDa) in hGC cells

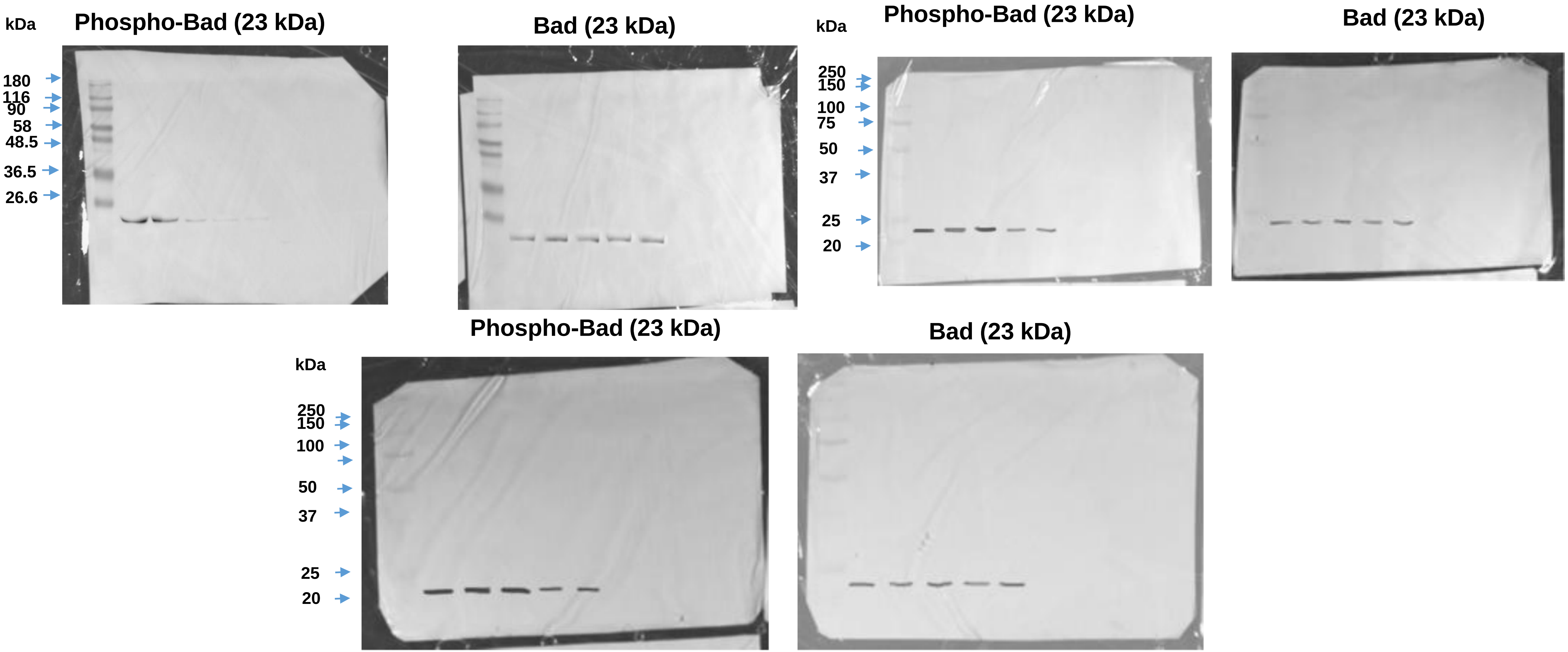

Revelation of the blots performed with the a G-box SynGene (Ozyme, St Quentin en Yvelines, France) and GeneSnap software : a composite with the membrane with the markers and the membrane revealed with ECL after the antibodies incubation is shown**.** Membranes were probed with phospho-Bad antibodies and then after stripping they were probed with total Bad antibodies.

Supplemental figure 10

StAR (32 kDa) in KGN cells

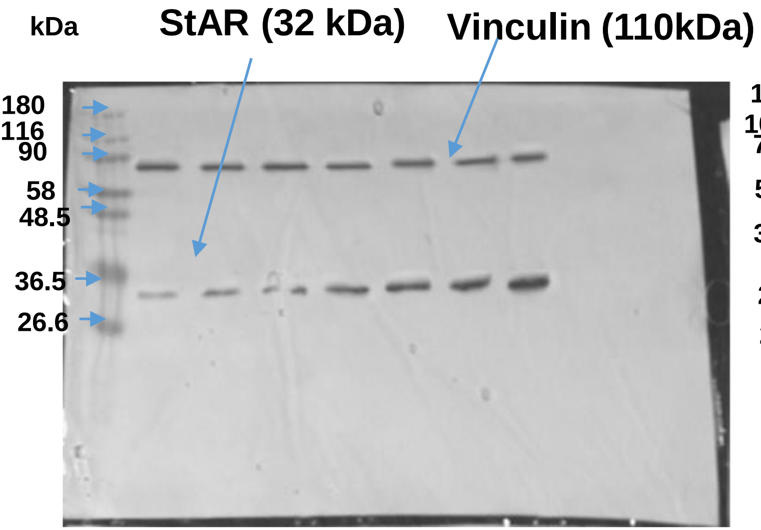

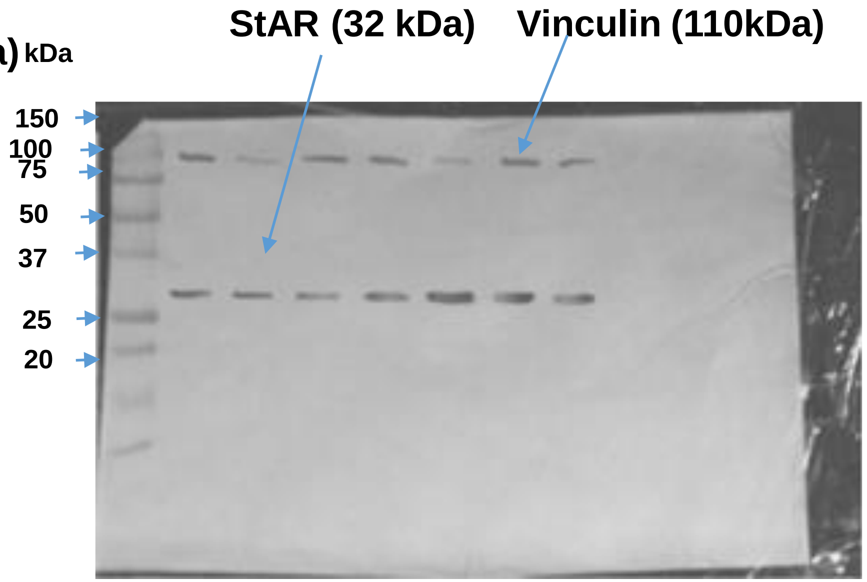

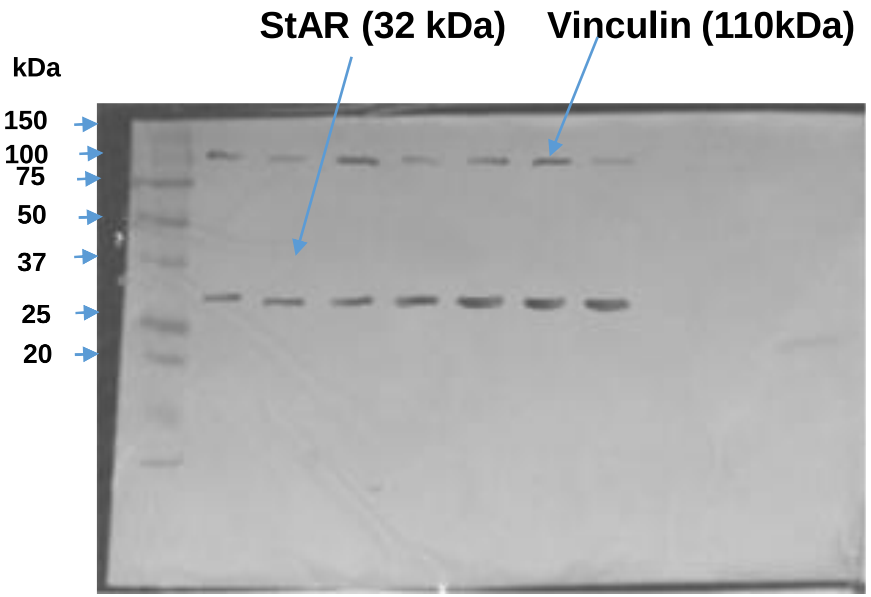

Revelation of the blots performed with the a G-box SynGene (Ozyme, St Quentin en Yvelines, France) and GeneSnap software : a composite with the membrane with the markers and the membrane revealed with ECL after the antibodies incubation is shown**.** Membranes were probed with StAR and Vinculin antibodies.

Supplemental figure 11

StAR (32 kDa) in hGC cells

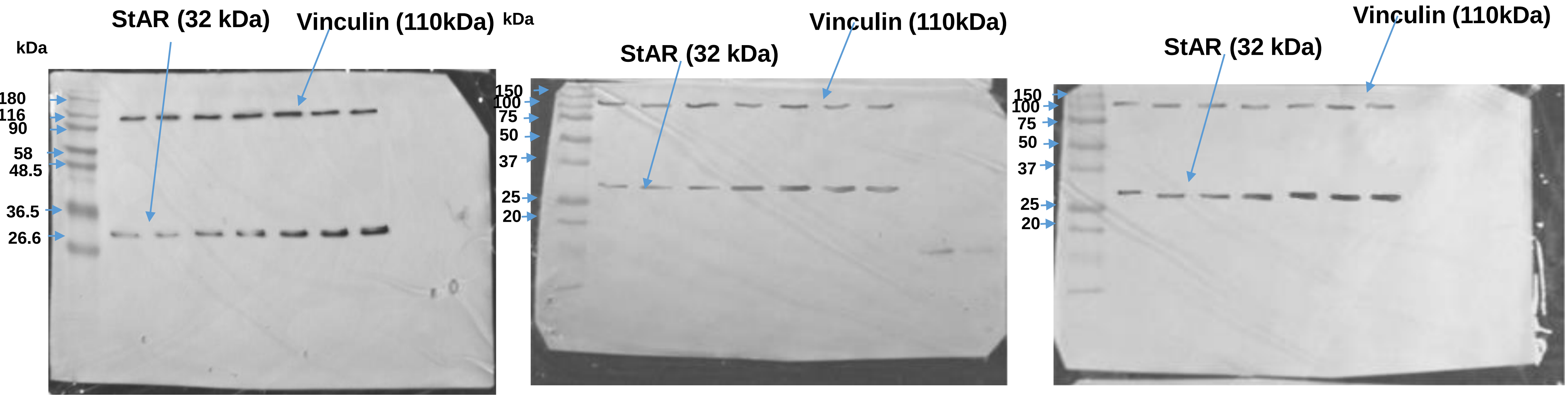

Revelation of the blots performed with the a G-box SynGene (Ozyme, St Quentin en Yvelines, France) and GeneSnap software : a composite with the membrane with the markers and the membrane revealed with ECL after the antibodies incubation is shown**.** Membranes were probed with StAR and Vinculin antibodies.

Supplemental figure 12

Phospho-CREB (43 kDa) in KGN cells

Phospho-CREB (43 kDa)

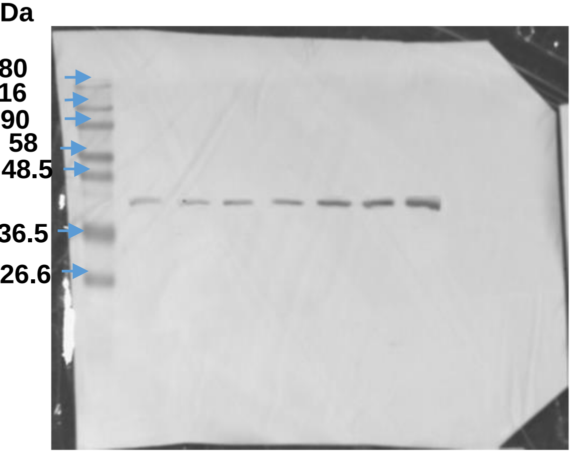

CREB (43 kDa)

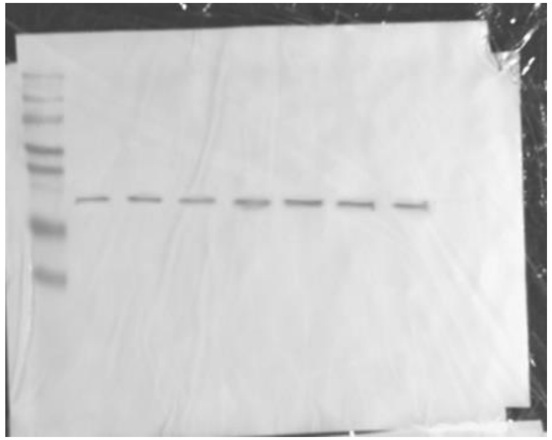

Phospho-CREB (43 kDa)

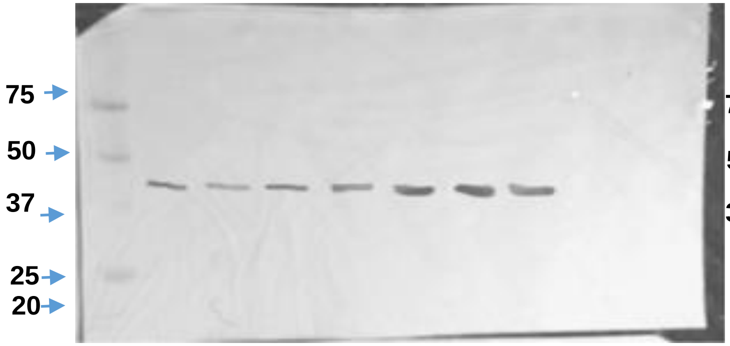

CREB (43 kDa)

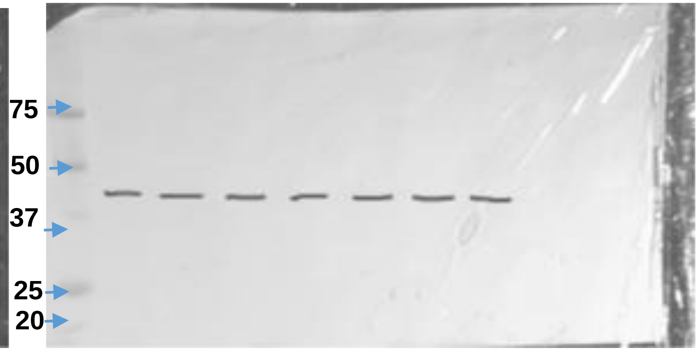

Phospho-CREB (43 kDa)

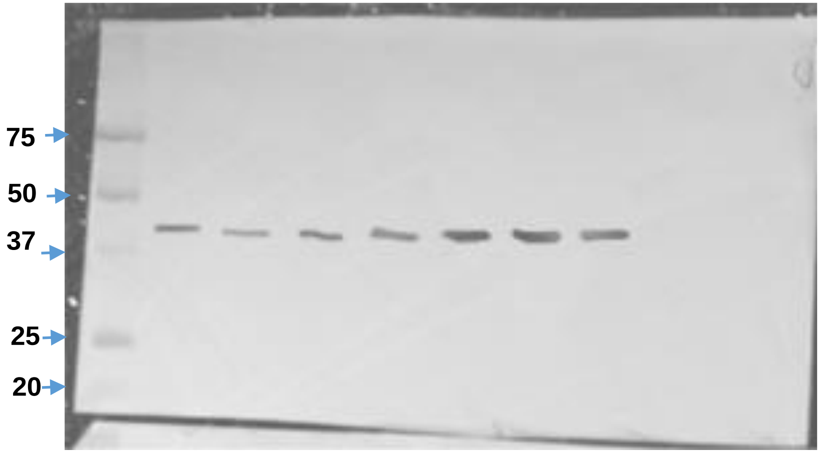

CREB (43 kDa)

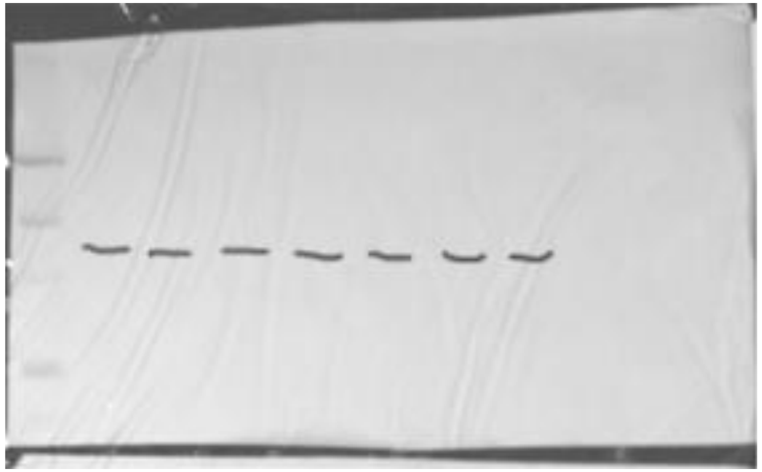

Revelation of the blots performed with the a G-box SynGene (Ozyme, St Quentin en Yvelines, France) and GeneSnap software : a composite with the membrane with the markers and the membrane revealed with ECL after the antibodies incubation is shown**.** Membranes were probed with phospho-Creb antibodies and then after stripping they were probed with with total Creb antibodies.

Supplemental figure 13

Phospho-CREB (43 kDa) in hGC cells

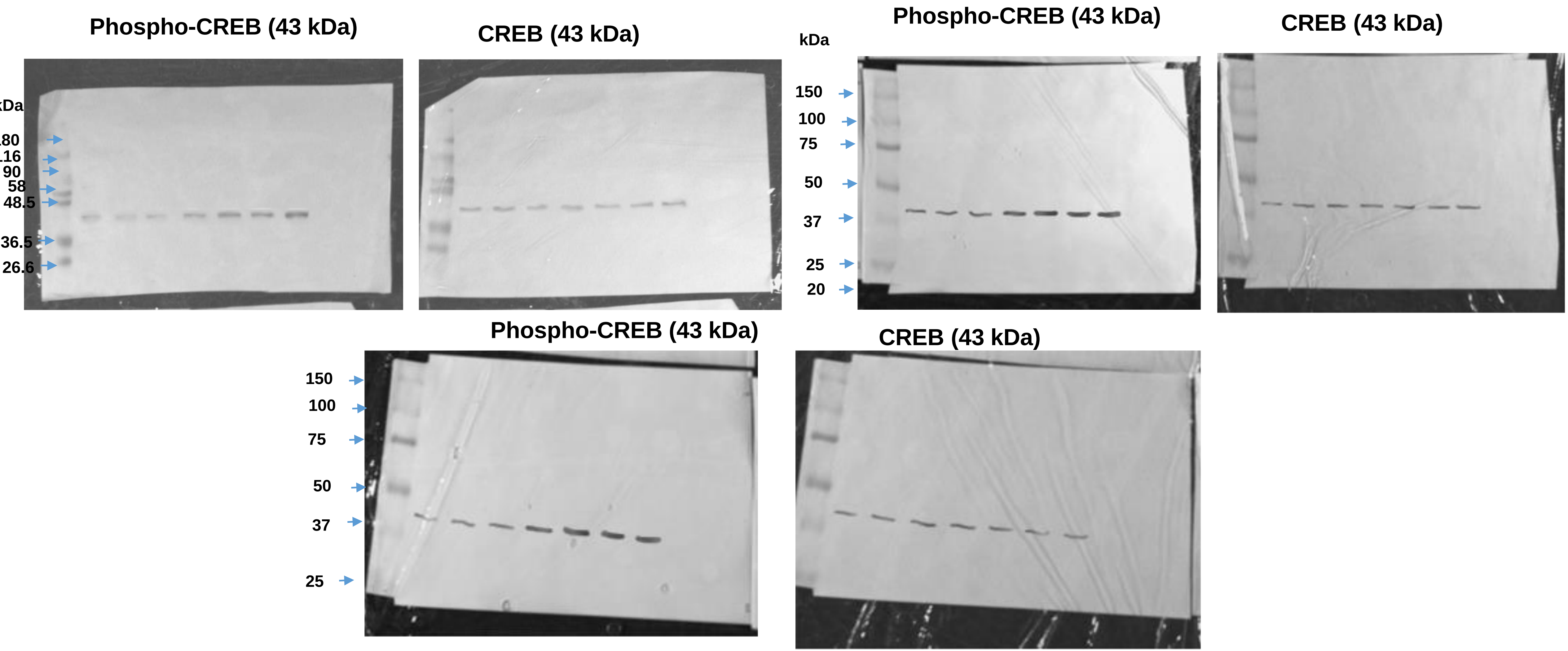

Revelation of the blots performed with the a G-box SynGene (Ozyme, St Quentin en Yvelines, France) and GeneSnap software : a composite with the membrane with the markers and the membrane revealed with ECL after the antibodies incubation is shown**.** Membranes were probed with phospho-Creb antibodies and then after stripping they were probed with with total Creb antibodies.

Supplemental figure 14

Phospho-AKT (56 kDa) in KGN cells

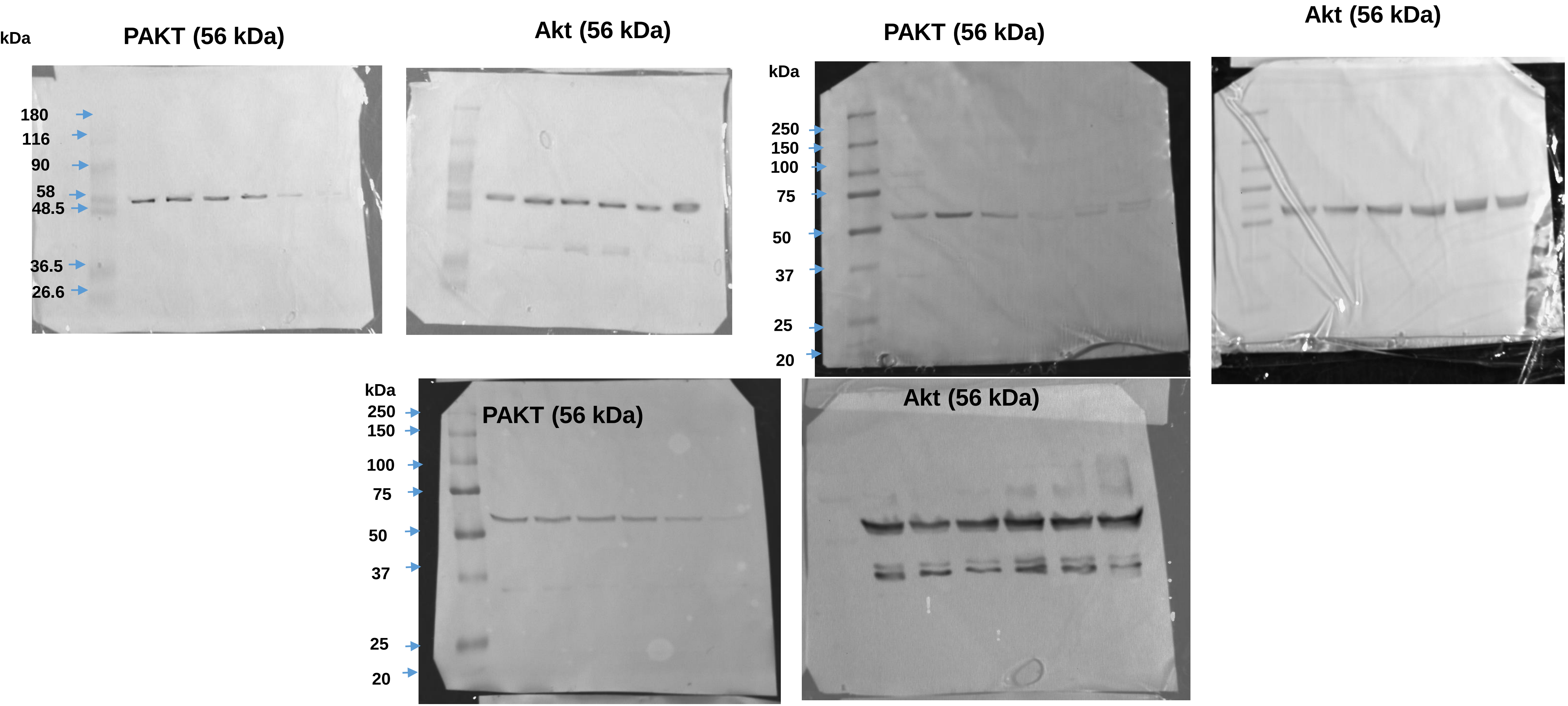

Revelation of the blots performed with the a G-box SynGene (Ozyme, St Quentin en Yvelines, France) and GeneSnap software : a composite with the membrane with the markers and the membrane revealed with ECL after the antibodies incubation is shown**.** Membranes were probed with phospho-Akt antibodies and then after stripping they were probed with with total Akt antibodies.

Supplemental figure 15

Phospho-AKT (56 kDa) in hGC cells

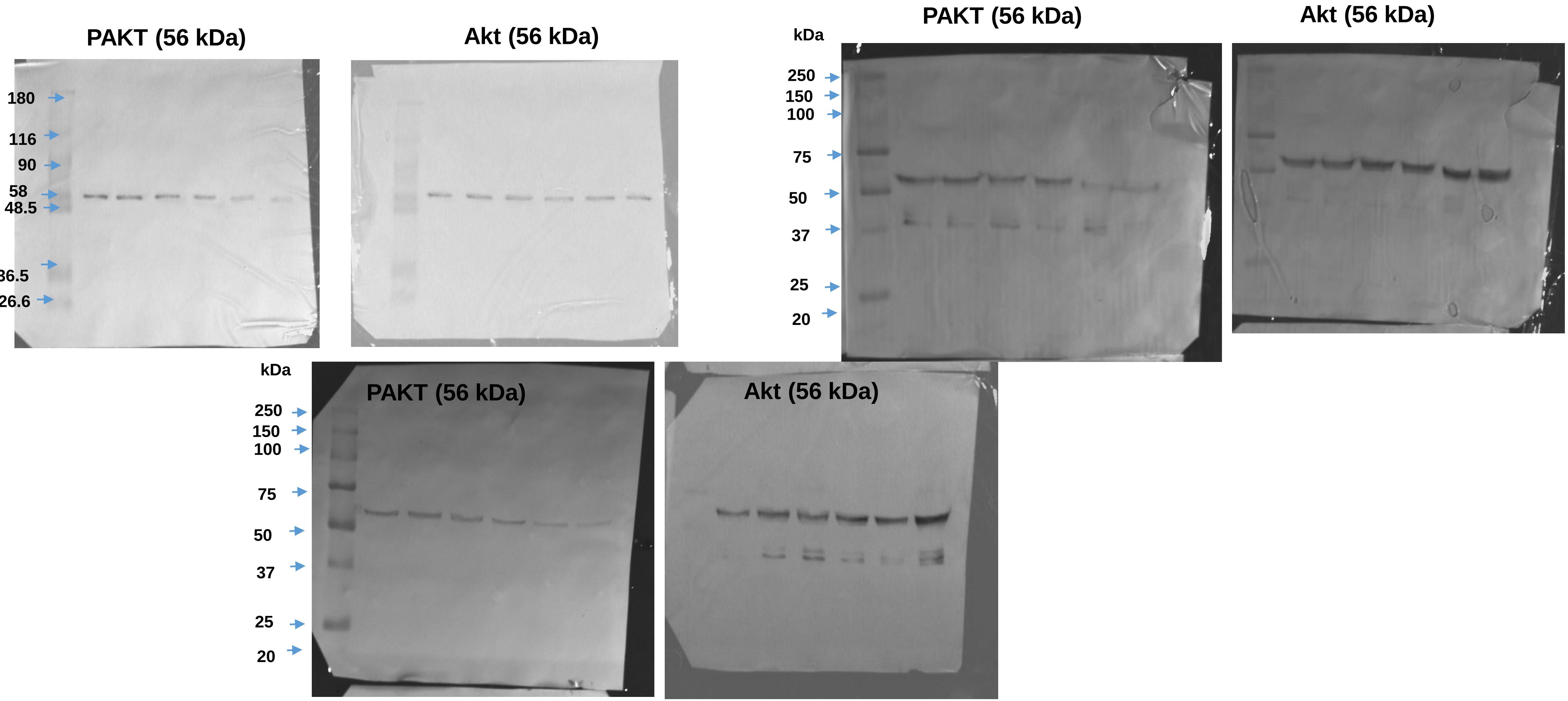

Revelation of the blots performed with the a G-box SynGene (Ozyme, St Quentin en Yvelines, France) and GeneSnap software : a composite with the membrane with the markers and the membrane revealed with ECL after the antibodies incubation is shown**.** Membranes were probed with phospho-Akt antibodies and then after stripping they were probed with with total Akt antibodies.

Supplemental figure 16

pERK1/2 (42/44 kDa) in KGN cells

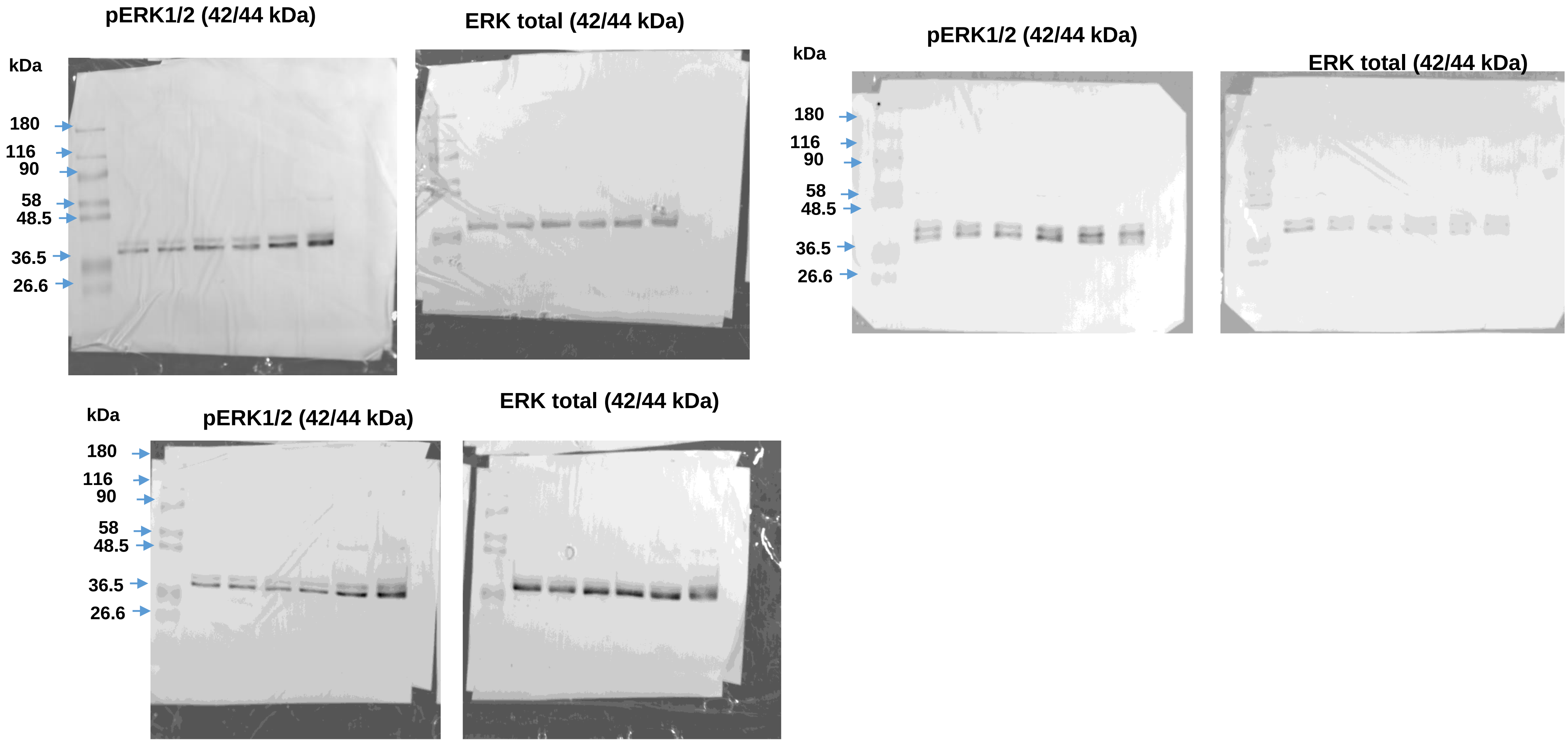

Revelation of the blots performed with the a G-box SynGene (Ozyme, St Quentin en Yvelines, France) and GeneSnap software : a composite with the membrane with the markers and the membrane revealed with ECL after the antibodies incubation is shown**.** Membranes were probed with phospho-ERK1/2 antibodies and then after stripping they were probed with with total ERK1/2 antibodies.

Supplemental figure 17

pERK1/2 (42/44 kDa) in hGC cells

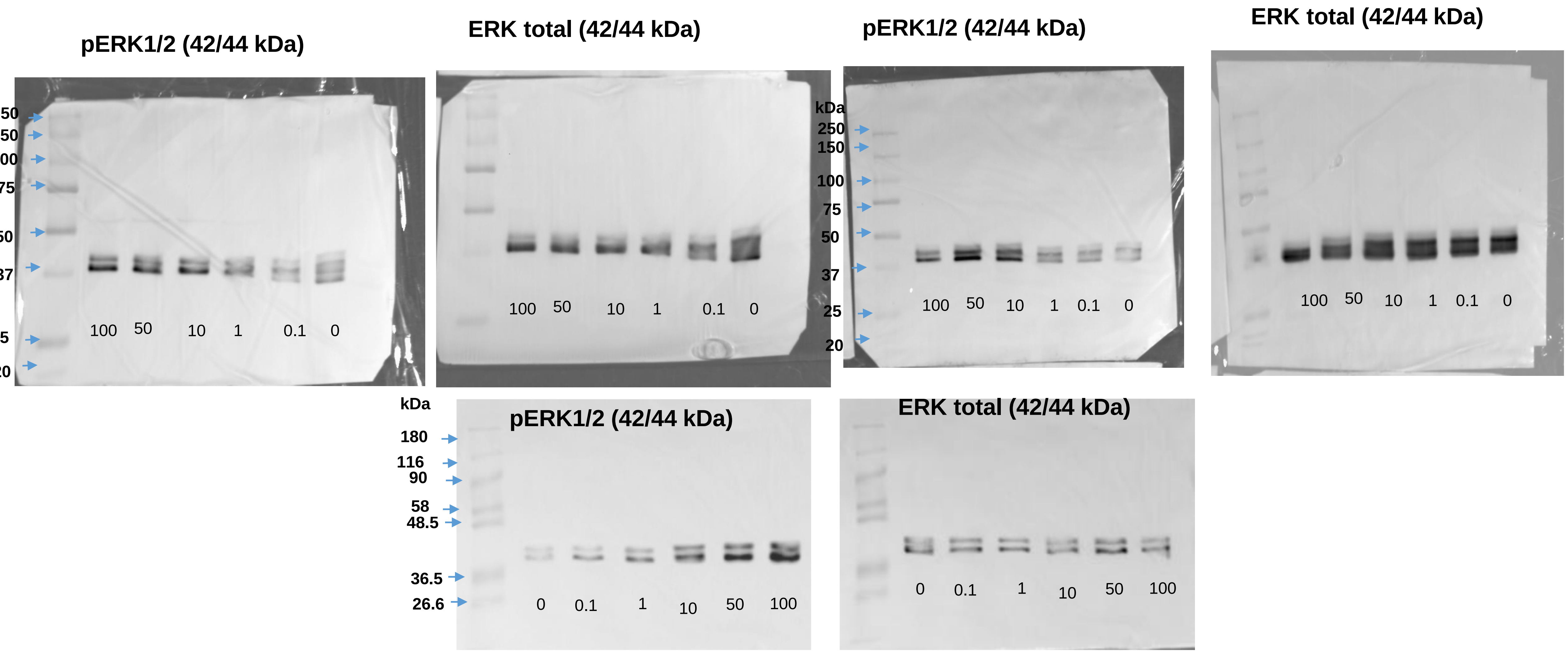

Revelation of the blots performed with the a G-box SynGene (Ozyme, St Quentin en Yvelines, France) and GeneSnap software : a composite with the membrane with the markers and the membrane revealed with ECL after the antibodies incubation is shown**.** Membranes were probed with phospho-ERK1/2 antibodies and then after stripping they were probed with with total ERK1/2 antibodies.

Supplemental figure 18
